# A comprehensive data mining study shows that most nuclear receptors act as newly proposed homeostasis-associated molecular pattern receptors

**DOI:** 10.1186/s13045-017-0526-8

**Published:** 2017-10-24

**Authors:** Luqiao Wang, Gayani Nanayakkara, Qian Yang, Hongmei Tan, Charles Drummer, Yu Sun, Ying Shao, Hangfei Fu, Ramon Cueto, Huimin Shan, Teodoro Bottiglieri, Ya-feng Li, Candice Johnson, William Y. Yang, Fan Yang, Yanjie Xu, Hang Xi, Weiqing Liu, Jun Yu, Eric T. Choi, Xiaoshu Cheng, Hong Wang, Xiaofeng Yang

**Affiliations:** 1 Department of Cardiovascular Medicine, The Second Affiliated Hospital of Nanchang University, Nanchang, Jiangxi 330006 China; 2 Center for Metabolic Disease Research, Lewis Katz School of Medicine at Temple University, Philadelphia, PA 19140 USA; 3 Centers for Cardiovascular Research and Thrombosis Research, Lewis Katz School of Medicine at Temple University, Philadelphia, PA 19140 USA; 4 Department of Pharmacology, Lewis Katz School of Medicine at Temple University, Philadelphia, PA 19140 USA; 5 Department of Surgery, Lewis Katz School of Medicine at Temple University, Philadelphia, PA 19140 USA; 6 Department of Cardiovascular Medicine, The First Affiliated Hospital of Kunming Medical University, Kunming, Yunnan 650032 China; 7 Department of Ultrasound, Xijing Hospital and Fourth Military Medical University, Xi’an, Shaanxi 710032 China; 8 Department of Pathophysiology, Zhongshan School of Medicine, Sun Yat-sen University, Guangzhou, Guangdong 510080 China; 9 Institute of Metabolic Disease, Baylor Research Institute, 3500 Gaston Avenue, Dallas, TX 75246 USA

**Keywords:** Nuclear receptors (NRs), Homeostasis-associated molecular pattern receptors, Atherosclerosis, Metabolic disease, Cardiovascular disease

## Abstract

**Background:**

Nuclear receptors (NRs) can regulate gene expression; therefore, they are classified as transcription factors. Despite the extensive research carried out on NRs, still several issues including (1) the expression profile of NRs in human tissues, (2) how the NR expression is modulated during atherosclerosis and metabolic diseases, and (3) the overview of the role of NRs in inflammatory conditions are not fully understood.

**Methods:**

To determine whether and how the expression of NRs are regulated in physiological/pathological conditions, we took an experimental database analysis to determine expression of all 48 known NRs in 21 human and 17 murine tissues as well as in pathological conditions.

**Results:**

We made the following significant findings: (1) NRs are differentially expressed in tissues, which may be under regulation by oxygen sensors, angiogenesis pathway, stem cell master regulators, inflammasomes, and tissue hypo-/hypermethylation indexes; (2) NR sequence mutations are associated with increased risks for development of cancers and metabolic, cardiovascular, and autoimmune diseases; (3) NRs have less tendency to be upregulated than downregulated in cancers, and autoimmune and metabolic diseases, which may be regulated by inflammation pathways and mitochondrial energy enzymes; and (4) the innate immune sensor inflammasome/caspase-1 pathway regulates the expression of most NRs.

**Conclusions:**

Based on our findings, we propose a new paradigm that most nuclear receptors are anti-inflammatory homeostasis-associated molecular pattern receptors (HAMPRs). Our results have provided a novel insight on NRs as therapeutic targets in metabolic diseases, inflammations, and malignancies.

**Electronic supplementary material:**

The online version of this article (10.1186/s13045-017-0526-8) contains supplementary material, which is available to authorized users.

## Background

Pathogen-associated molecular patterns (PAMPs) and danger-associated molecular patterns (DAMPs) generated during microbial invasion or tissue injury act as stimuli and activate the innate immune system to respond to infection or injury [1]. The key cellular receptors that recognize the “threat” signals initiated by PAMPs and DAMPs are referred to as PRRs (pattern recognition receptors). One of the receptor families that are highly characterized as PRRs is the Toll-like receptor (TLR) family. Most of the TLRs are mainly located on the plasma membrane and activate inflammatory genes to counteract tissue injury and mediate repair. Moreover, TLRs work in synergy with cytosolic PRR families like NLRs (NOD (nucleotide-binding oligomerization domain)-like receptors) to recognize DAMPs, particularly in what we proposed—inflammation-privileged tissues where inflammasome component genes that initiate inflammation are not constitutively expressed [2, 3]. Additionally, four other PRR families including C-type lectin receptors, retinoid acid-inducible gene 1 (RIG-1), absent in melanoma-2 (AIM-2), and receptor for advanced glycation end products (RAGE, also a receptor for high-mobility group box 1 (HMGB1)) have also been characterized [4].

Previously, using endogenous metabolite lysophospholipids (LPLs) as a prototype, we proposed a new paradigm for the first time that certain metabolites that play cellular functions during normal physiological status can adapt as pro-inflammatory mediators at elevated concentrations. We named such metabolites as “conditional DAMPs” and their endogenous receptors as “conditional DAMP receptors.” We further pointed out significant loopholes in the current danger model which identify only the six receptors mentioned above as PRRs, which we named as “classical DAMP receptors” [5]. Along the line, we recently reported a series of significant findings on the expression and roles of caspase-1 in the NLR pathway in vascular inflammation [2, 6–15]. In the same publication mentioned above, we concluded that activation of inflammation by conditional DAMPs may be realized via binding to their own intrinsic receptors and may not necessarily always involve or “converge to” TLRs, NLRs, and other classical DAMP receptors [5].

Another significant problem associated with the current danger theory is that it fails to recognize the roles played by potential endogenous metabolites in anti-inflammatory responses, inflammation resolution, and maintenance of homeostasis. Therefore, we further advanced the current paradigm by proposing endogenous metabolites such as lysophosphatidylserine and lysophosphatidylethanolamine that not only maintain homeostasis at physiological levels, but also act as anti-inflammatory mediators to inhibit inflammation and promote inflammation resolution at pathologically elevated levels as homeostasis-associated molecular patterns (HAMPs). Furthermore, we proposed that these HAMPs bind to their receptors (HAMP receptors) to initiate anti-inflammatory/homeostatic signaling and promote inflammation resolution [5]. However, an outstanding issue of whether endogenous lipophilic metabolites that bind to nuclear receptors can serve as HAMPs remains unknown.

The nuclear hormone receptor superfamily has 48 lipophilic ligand-activated receptors including 32 nuclear hormone receptors (NHRs) for thyroid and steroid hormones, retinoids, and vitamin D, as well as 16 orphan nuclear receptors where the ligands are yet unknown [16–18]. Nuclear receptors (NRs), as transcription factors, have the ability to directly bind to DNA and regulate the expression of adjacent genes [19, 20]. Ligands for some of these NRs have been recently identified, including lipid metabolites such as fatty acids, prostaglandins, or cholesterol derivatives. These ligands can regulate gene expression by binding to NRs [21]. Ligand binding to a NR results in a conformational change and activation of the receptor, leading to up- or downregulation of the target gene expression. Thus, NRs are involved in the regulation of various physiological processes including development, homeostasis, and metabolism of the organism [22] and pathogenesis of metabolic disease in response to metabolic/environmental changes [23].

However, despite the recent progress, there are many aspects of NRs that have not yet been explored: *first*, the expression profile of NRs under physiological conditions in various human tissues have not been studied; *second*, whether the expression of certain NRs are either upregulated or downregulated in atherogenic and metabolic disease-related pathological conditions are not clear; *third*, mechanistically, whether pro-/anti-inflammatory signaling is negatively/positively associated with the expression of NRs is not known; and *fourth*, whether NRs have the capacity to function as our newly proposed HAMP receptors, which suppress inflammatory responses and maintain tissue homeostasis in response to the stimulation of exogenous and endogenous PAMPs/DAMPs. To address these questions, we took a “panoramic view” at the tissue expression pattern of all 48 identified human and mouse NRs. Our results demonstrated that NRs are differentially expressed among tissues at physiological conditions, which may be regulated by oxygen sensors, vascular endothelial growth factor pathways, stem cell master regulators, innate immune sensors, and DNA hypo-/hypermethylation status. We also found that the expressions of certain NRs have less tendency to be upregulated than to be downregulated in atherogenic conditions, metabolic diseases, which may be contributed by significant regulation of innate immune sensor caspase-1/inflammasome pathway. Our findings provide novel insights into the upstream regulation of nuclear receptors in physiological, autoimmune arthritis, and cardiovascular and metabolic disease conditions.

## Methods

### Tissue expression profiles of genes encoding nuclear receptors

**Fig. 1:**
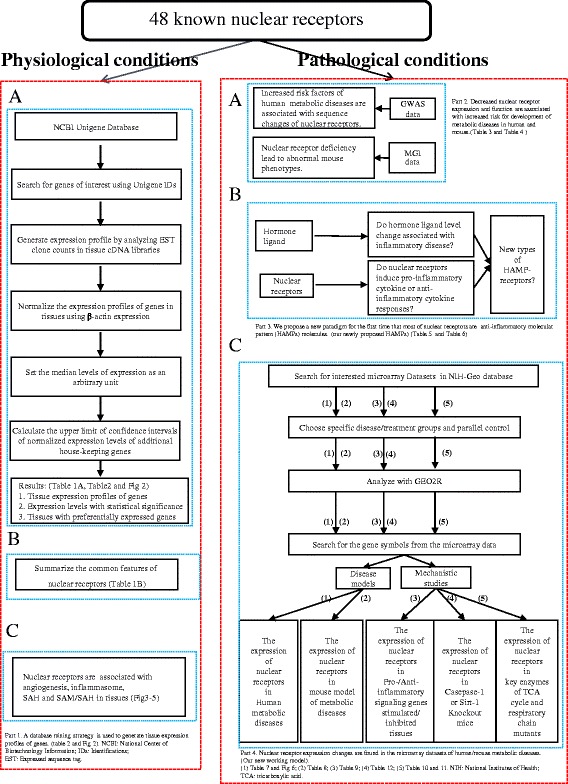
Flow chart of database mining strategy and two parts of data organization. Part1: shows the database mining strategy utilized to generate tissue nuclear receptor expression profile. Part 2: the strategy utilized to measure nuclear receptor expression in human and mouse metabolic diseases. Parts 3 and 4: shows the strategy that is used to analyze the microarray data sets and identifying nuclear receptors as homeostasis-associated molecular pattern receptors (HAMPRs)

An experimental data mining strategy (Fig. 1) was used to analyze the expression profiles of mRNA transcripts of NR genes in 21 different human and 17 mouse tissues including the heart and vasculature. We utilized an experimentally verified mRNA expression in the expressed sequence tag (EST) databases of the National Institutes of Health (NIH)/National Center of Biotechnology Information (NCBI) UniGene (http://www.ncbi.nlm.nih.gov/sites/entrez?db=unigene) to determine the transcription profile of nuclear receptors in tissues of interest. Transcripts per million of genes of interest were normalized to that of housekeeping gene β-actin in each given tissue to calculate the arbitrary units of gene expression. A confidence interval of the expression variation of housekeeping genes was generated by calculating the mean plus two times that of the standard deviation of the arbitrary units of three randomly selected housekeeping genes (PRS27A, GADPH, and ARHGDIA in human; Ldha, Nono, and Rpl32 in mouse) normalized by β-actin in the given tissues. If the expression variation of a given gene in the tissues was larger than the upper limit of the confidence interval, the high expression levels of genes in the tissues were considered statistically significant. Gene transcripts where the expression level was lower than one per million were technically considered as no expression.

### Expression profiles of nuclear receptors in disease models and cell activity

Microarray datasets were collected from the Array Express of European Bioinformatics Institute, which stores data from high-throughput functional genomics experiments (https://www.ebi.ac.uk/arrayexpress). These data include the information of the expression of nuclear receptors through experiments submitted directly to Array Express or imported from the NCBI Gene Expression Omnibus database. We used data from the following databases: (1) Metabolic disease: (a) adipose tissue and liver in high fat diet-induced obese mouse model versus normal diet controls, (b) aortic arch segment of the atherogenic apolipoprotein E gene knockout (apolipoprotein E (ApoE−/−)) mice versus wild-type mouse aorta controls, (c) pancreatic islets and white fat of leptin receptor mutant *db/db* type II diabetic mice versus control mice, (d) oxidized low-density lipoprotein (Ox-LDL)-stimulated mouse endothelial cells versus control endothelial cells, and (e) high-concentration homocysteine (Hcy)-treated human aortic smooth muscle cells (HASMCs) versus low-concentration homocysteine (Hcy)-treated vascular smooth muscle cells (VSMCs); (2) CD4+Foxp3+ regulatory T cell (Treg) polarization/differentiation—we examined the expression changes of the nuclear receptors in Tregs versus effector T cells in mice, as well as in vitro, with cytotoxic T-lymphocyte-associated protein 4 (CTLA-4) ligation; (3) mRNA expression of NR changes due to the stimulus with pro-/anti-inflammation conditions; and (4) we screened the datasets among energy metabolic nuclear receptors of tricarboxylic acid (TCA) cycle and respiratory chain. The modulation of nuclear receptor expression in cancers were determined by analyzing the Cancer Genome Atlas database.

### Application of big GWAS data to clarify the relationship between nuclear receptors and metabolic disease

Genome-wide association studies (GWASs) continue to be a widely used approach to detect genetic association with a phenotype of interest in well-defined populations. Various anthropometric measures serve as surrogates for obesity, with body mass index (BMI) (HGVPM 1111 and 564) and waist-hip ratio (HGVPM 1114) as the most frequently used markers in epidemiologic studies aimed at assessing obese disease risks. Anti-cyclic citrullinated peptide-positive rheumatoid arthritis status (HGVPM 38) and rheumatoid arthritis (HGVPM 235) are the most frequently used markers in epidemiologic studies aimed at rheumatoid arthritis risk. Fasting plasma glucose (HGVPM 825), homeostatic model assessment of β-cell function (HGVPM 827), fasting insulin (HGVPM 822), homeostatic model assessment of insulin resistance (HGVPM 826), glycated hemoglobin levels (HGVPM 1081), glycosylated hemoglobin (HGVPM 569), 2-h glucose challenge (HGVPM 769), type II diabetes status (HGVPM 4 and 5), early onset type II diabetes mellitus (HGVPM 74), proinsulin levels (HGVPM 1538), and a-glucose (HGVPM 3639) are the most frequently used markers in epidemiologic studies aimed at diabetes risks. Serum cholesterol (HGVPM 568), lipids (CH3) (HGVPM 3602), lipids (CH2) (HGVPM 3611), lipids (CH2CO) (HGVPM 3616), lipids (CH=CH*CH2CH2) (HGVPM 3640), and systolic blood pressure (HGVPM 563) are the most frequently used markers in epidemiologic studies aimed at vascular atherosclerosis risks. With the advent of large GWASs, we now have the ability to identify NRs associated with dangerous risks for specific disease.

### Application of MGI data to clarify the abnormal mouse phenotypes in nuclear receptor knockout mouse adipose, cardiovascular, metabolism, and endocrine systems

MouseMine (www.informatics.jax.org) is a new data warehouse for accessing mouse data from Mouse Genome Informatics (MGI). The main source of MouseMine data is MGI, which includes a wealth of information about the structure and function of the mouse genome, developmental gene expression patterns, phenotypic effects caused by mutations, and annotations of human disease models. The “Human-mouse: disease connection” tool (www.informatics.jax.org/humanDisease.shtml) supports uploading a list of nuclear receptor gene IDs/symbols and getting back certain information about those nuclear receptors, such as those associated human diseases and abnormal mouse phenotypes reported in adipose, cardiovascular, metabolic, and endocrine systems.

### Tissue SAH and SAM measurements in mice

The concentrations of S-adenosyl methionine (SAM) and S-adenosyl homocysteine (SAH) were measured in six tissues (heart, liver, lung, kidney, spleen, and brain) in C57BL/6J (*n* = 4) mice from 13.4 to 18 weeks of age. Mouse tissues were collected and homogenized in 0.4 mol/L perchloric acid (PCA) solution. The homogenized tissues were centrifuged for 10 min at 2000 rpm. Supernatant was collected and stored at −80 °C. SAM and SAH levels were analyzed by liquid chromatography-electrospray ionization-tandem mass spectrometry (LC-ESI-MS/MS; Institute of Metabolic Disease, Baylor Research Institute, Dallas, TX). The unit of SAH level in tissues is nanomole per gram [24].

## Results

### Nuclear receptors are differentially expressed in tissues. Nuclear receptor expression is associated with angiogenesis pathway, stem cell master genes, PRRs, and tissue hypomethylation/hypermethylation indices

As summarized in Table 1, the NR superfamily includes 48 NRs classified into seven families, such as class I-thyroid hormone receptor-like family (19 members), class II-retinoid X receptor-like family (12 members), class III-estrogen receptor-like family (9 members), class IV-nerve growth factor IB-like family (3 members), class V-steroidogenic factor-like receptor family (2 members), class VI-germ cell nuclear receptor-like family (1 member), and class O-miscellaneous family (2 members). In addition, we summarized seven common features of the NR superfamily in Table 2. One of the most striking features of NRs is that in addition to transduce steroid, thyroid, retinoid, and other hormone signals, NRs can also serve as metabolic sensors and xenobiotic sensors for high-affinity ligands and low-affinity molecular patterns [25]. Several reports showed that NRs not only bind to specific ligands but also recognize structural patterns (Table 3), which raises a possibility for NRs to recognize many endogenous metabolites that can act as HAMPs that are yet to be identified/characterized [5].

**Table 1:** The UniGene ID of 48 human nuclear receptors and mouse homologs

Gene name (full name)		NRNC symbol	Receptor	Ligand(s)	ID	
					Human	Mouse (Mm.)
					(Hs.)	
Class I—thyroid hormone receptor-like						
THRA	Thyroid hormone receptor, alpha	NR1A1	Thyroid hormone receptor	Thyroid hormone	724	265917
THRB	Thyroid hormone receptor, beta	NR1A2			187861	32563
RARA	Etinoic acid receptor, alpha	NR1B1	Retinoic acid receptor	Vitamin A and related compounds	654583	439744
RARB	Etinoic acid receptor, beta	NR1B2			654490	259318
RARG	Etinoic acid receptor, gamma	NR1B3			1497	1273
PPARA	Peroxisome proliferator-activated receptor alpha	NR1C1	Peroxisome proliferator-activated receptor	Fatty acids, prostaglandins	103110	212789
PPARD	Peroxisome proliferator-activated receptor delta	NR1C2			696032	328914
PPARG	Peroxisome proliferator-activated receptor gamma	NR1C3			162646	3020
NR1D1	Nuclear receptor subfamily 1 group D member 1	NR1D1	Rev-ErbA	Heme	592130	390397
NR1D2	Nuclear receptor subfamily 1 group D member 2	NR1D2			37288	26587
RORA	RAR-related orphan receptor A	NR1F1		Cholesterol	560343	427266
RORB	RAR-related orphan receptor B	NR1F2			494178	234641
RORC	RAR-related orphan receptor C	NR1F3			256022	4372
NR1H3	Nuclear receptor subfamily 1 group H member 3	NR1H3	Liver X receptor-like receptor	Oxysterols	438863	22690
NR1H2	Nuclear receptor subfamily 1 group H member 2	NR1H2			432976	968
NR1H4	Nuclear receptor subfamily 1 group H member 4	NR1H4			282735	3095
VDR	Vitamin D (1,25-dihydroxyvitamin D3) receptor	NR1I1	Vitamin D receptor-like receptor	Vitamin D	524368	245084
NR1I2	Nuclear receptor subfamily 1group I member 2	NR1I2		Xenobiotics	7303	8509
NR1I3	Nuclear receptor subfamily 1 group I member 3	NR1I3		Androstane	349642	486506
Class II—retinoid X receptor-like						
HNF4A	Hepatocyte nuclear factor 4, alpha	NR2A1	Hepatocyte nuclear factor-4 receptor	Fatty acids	116462	202383
HNF4G	Hepatocyte nuclear factor 4, gamma	NR2A2			241529	330897
RXRA	Retinoid X receptor alpha	NR2B1	Retinoid X receptor	Retinoids	590886	24624
RXRB	Retinoid X receptor beta	NR2B2			388034	1243
RXRG	Retinoid X receptor gamma	NR2B3			26550	3475
NR2C1	Nuclear receptor subfamily 2 group C member 1	NR2C1	Testicular receptor	UD	108301	107483
NR2C2	Nuclear receptor subfamily 2 group C member 1	NR2C2			555973	87062
NR2E1	Nuclear receptor subfamily 2 group E member 1	NR2E1	Tailless-like receptors	UD	157688	287100
NR2E3	Nuclear receptor subfamily 2 group E member 3	NR2E3			187354	103641
NR2F1	Nuclear receptor subfamily 2 group F member 1	NR2F1	COUP-TF-like receptors	UD	347991	439653
NR2F2	Nuclear receptor subfamily 2 group F member 2	NR2F2			519445	158143
NR2F6	Nuclear receptor subfamily 2 group F member 6	NR2F6			466148	28989
Class III—estrogen receptor-like						
ESR1	Estrogen receptor 1	NR3A1	Estrogen receptor	Estrogens	208124	9213
ESR2	Estrogen receptor 2	NR3A2			660607	2561
ESRRA	Estrogen-related receptor alpha	NR3B1	Estrogen-related receptor	UD	110849	386776
ESRRB	Estrogen-related receptor beta	NR3B2			435845	235550
ESRRG	Estrogen-related receptor gamma	NR3B3			444225	89989
NR3C1	Nuclear receptor subfamily 3 group C member 1	NR3C1	3-Ketosteroid receptors	Cortisol	122926	129481
NR3C2	Nuclear receptor subfamily 3 group C member 2	NR3C2		Aldosterone	163924	324393
PGR	Progesterone receptor	NR3C3		Progesterone	32405	12798
AR	Androgen receptor	NR3C4		Testosterone	76704	439657
Class IV—nerve growth factor IB-like						
NR4A1	Nuclear receptor subfamily 4 group A member 1	NR4A1	Nerve growth factor IB-like receptors	UD	524430	119
NR4A2	Nuclear receptor subfamily 4 group A member 2	NR4A2			563344	3507
NR4A3	Nuclear receptor subfamily 4 group A member 3	NR4A3			279522	247261
Class V—steroidogenic factor-like						
NR5A1	Nuclear receptor subfamily 5 group A member 1	NR5A1	Fushi tarazu F1-like receptors	Phosphatidylinositols	495108	31387
NR5A2	Nuclear receptor subfamily 5 group A member 2	NR5A2			33446	16794
Class VI—germ cell nuclear factor-like						
NR6A1	Nuclear receptor subfamily 6 group A member 1	NR6A1	Germ cell nuclear factor receptors	UD	586460	439703
Class O—miscellaneous						
NR0B1	Nuclear receptor subfamily 0 group B member 1	NR0B1	DAX-like Receptors	UD	268490	5180
NR0B2	Nuclear receptor subfamily 0 group B member 2	NR0B2			427055	346759

*UD* undetermined

**Table 2:** The common features of nuclear receptors

Common features of nuclear receptors	PMID
1. Five domain structures including N-terminal regulatory domain, DNA binding domain, hinge region, ligand-binding domain, and C-terminal domain	10406480/10751636/12893880
2. Lipophilic ligand-activated transcription factors including orphan receptors for unknown endogenous ligands	8,807,884/10671476
3. 48 known super human family members including seven groups, mice (49), rats (47), C. elephant (270)	10219237/9460643/15059999
4. 350 co-regulators to facilitate their functions	22733267
5. Transduce steroid, thyroid, retinoid, and other hormonal signals	11729302/8521507
6. Metabolic sensors and xenobiotic sensors for high-affinity ligands and low-affinity molecular patterns	20615454
7. Serve as the targets for 13% FDA-approved drugs	17139284

**Table 3:** Nuclear receptors can recognize and bind many ligands which have similar structures/patterns via its ligand-binding domain

Features of nuclear receptors’ ligand-binding domain	PMID
1. Ligand-binding domains have the capacity to bind coactivator segments with LXXLL sequences, and corepressor segments with LXXXLXXX[I/L] sequences (where L = leucine, I = isoleucine, and X = any amino acid)	9808622
2. A single nuclear receptor controls the multitude of gene expressions	20148675
3. The ligand-binding domain consists of a hydrophobic pocket that can bind a hydrophobic ligand	20615454
4. Flexible ligands can contort to fit in the ligand-binding pocket	9501913
5. Pharmacological antagonists and have been shown to bind to the receptor in the ligand-binding site and to inhibit hormone-activated receptor function	
(1). NR1A1 ligand-binding domain can bind 3,5-dimethyl-3-isopropylthyronine except thyroid hormone	8523397
(2). NR1B3 ligand-binding domain can bind to all-trans retinoic acid except vitamin A and related compounds	7501014
(3). NR3A1 ligand-binding domain can bind to estradiol and raloxifene	9338790

To determine whether tissues have functional differences in sensing metabolic stressors and xenobiotic stressors via NRs, we hypothesized that various tissues express differential levels and certain types of NRs under physiological conditions. To examine this hypothesis, the expression of 48 NR genes in 21 human tissues and 17 mouse tissues were examined (fewer mouse tissues were examined due to unavailability of gene expression data for four types of mouse tissues, i.e., nerve, trachea, stomach, and vascular tissues in the NIH UniGene database) (Additional file 1: Figure S1). The results showed that some human tissues such as muscle (17), trachea (14), and nerve (10) express a large variety of NRs at high expression levels (Tables 4 and 5). This data suggests that the gene expression, differentiation, and function of these tissues may largely be regulated by NRs under normal physiological levels. Comparatively, eyes (7), adrenal gland (6), kidney (5), and adipose tissue (5) express more variety of NRs than the heart, liver, and pancreas (Table 4). Similarly, when comparing the human NR expression profile to that of the mouse, human tissues express much more types of NRs at high expression levels than mice. For example, although human and mouse muscles contain more variety of NRs at high levels relative to other tissues studied, human muscle expresses 17 NRs whereas mouse muscle expresses only 7 NRs. Among the 17 human muscle-expressed NRs, the higher expression of THRB, RORA, ESR1, ESRRA, NR3C2, and NR4A3 in human muscle is not seen in mouse muscle (Tables 4 and 5). Therefore, this indicates that these receptors were evolutionally gained, and addition of these NRs in humans may be responsible for the development of new muscle functions in response to environmental changes/nutritional changes that humans face. Furthermore, nearly half of the tissues examined (including the heart, liver, pancreas, brain, and lymph node) did not contain a large variety of NRs at high expression levels. These results suggested that the gene expression, differentiation, and function of these tissues may be largely dependent on those expressed NRs rather than the non-expressed NRs. Similarly, the human skin, spleen, stomach, vascular, blood, and lung tissue had minimal varieties of nuclear receptors in physiological conditions, since less than 4 out of 48 nuclear receptors are highly expressed (Additional file 2: Figure S2).

**Table 4:** 28 out of 43 nuclear receptors in classes I–IV are highly expressed in the human muscle, trachea, nerve, and other tissues

Gene	Human tissues															
	Adipose tissue	Adrenal gland	Brain	Eye	Heart	Intestine	Kidney	Liver	Lymph node	Muscle	Nerve	Pancreas	Skin	Spleen	Stomach	Trachea
Class I—thyroid hormone receptor-like (15 out of 19)																
THRA			*	*	*					*	*					
THRB										*						*
RARA																*
RARB				*			*	*								*
RARG																*
PPARA		*					*	*		*	*					
PPARD											*					*
PPARG	*					*					*					
NR1D1				*						*	*	*				*
NR1D2				*						*						
RORA		*								*			*			
RORC		*				*	*	*	*	*						*
NR1H3	*				*					*				*		
NR1H2										*						
VDR																*
Class II—retinoid X receptor-like (5 out of 12)																
RXRA										*						
RXRB																*
NR2C2									*	*						*
NR2F2	*			*						*	*	*				
NR2F6				*												
Class III—estrogen receptor-like (5 out of 9)																
ESR1		*								*						*
ESRRA							*		*	*		*				
NR3C2				*	*					*					*	*
PGR					*		*	*		*	*					
AR											*	*				*
Class IV—nerve growth factor IB-like (3 out of 3)																
NR4A1	*															
NR4A2		*	*								*					*
NR4A3	*	*								*	*					

*High expression

**Table 5:** 15 out of 41 nuclear receptors in classes I–VI are highly expressed in the mouse muscle, skin, and other tissues

Gene	Mouse tissues													
	Adrenal gland	Blood	Brain	Eye	Heart	Intestine	Kidney	Liver	Lung	Lymph node	Muscle	Pancreas	Skin	Spleen
Class I—thyroid hormone receptor-like (7 out of 19)														
Thra			*		*	*					*		*	
Rara										*		*	*	*
Ppara				*		*	*	*			*	*		*
Nr1d1	*										*			
Nr1d2	*										*			
Nr1h2									*				*	
Vdr													*	*
Class II—retinoid X receptor-like (5 out of 12)														
Rxra	*										*			
Nr2c1					*								*	
Nr2c2											*			
Nr2f2	*					*						*		
Nr2f6					*				*			*		
Class III—estrogen receptor-like (2 out of 9)														
Nr3c1											*		*	*
Ar		*												
Class VI—germ cell nuclear factor-like (1 out of 1)														
Nr6a1	*											*	*	*

*High expression

Based on the distribution pattern of highly expressed NRs among the tissues, we classified NRs into following four groups: very highly distributed, highly distributed, moderately distributed, and scarcely distributed (Table 6). In order to determine whether very highly distributed and highly distributed groups of NRs have any functional differences from that of moderately distributed and scarcely distributed group of NRs, we analyzed the potential signaling pathways with the Ingenuity Pathway Analyzer for these two major groups of NRs. The results in Table 6 show that among the top 10 pathways examined for each group, the two major NR groups share four signaling pathways such as FXR/retinoid X receptor (RXR) activation, hepatic cholestasis, aryl hydrocarbon receptor signaling, and RAR activation. The very highly distributed and highly distributed group of NRs have six specific top pathways including peroxisome proliferator-activated receptor (PPAR) signaling, glucocorticoid receptor signaling, melatonin signaling, estrogen receptor signaling, adipogenesis pathway, and PPARα/RARα activation. In contrast, the moderately distributed and scarcely distributed groups of NRs have another six specific top pathways including Oct4 stem cell pluripotency, pregnane X receptor (PXR)/RXR activation, LPS/IL-1-mediated inhibition of RXR function, retinoic acid-mediated apoptosis signaling, 25-dihydroxyvitamin D3 (vitamin D3) receptor (VDR)/RXR activation, and liver X receptor (LXR)/RXR activation. Of note, the NRs that have vitamin A, vitamin D, and retinoids as ligands are all included in the scarcely distributed group. Therefore, these data suggest that the tissue expression levels and distribution pattern of NRs can be used as an indicator of functional differences in tissues.

**Table 6:**
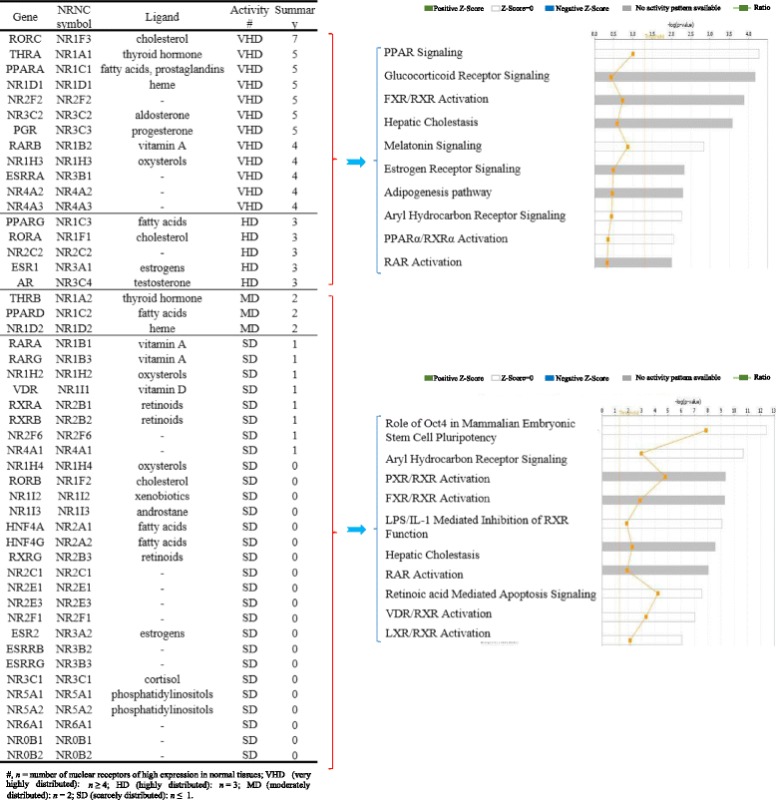
Nuclear receptors can be classified into four groups including very highly, highly, moderately, and scarcely distributed based on their distribution in tissues. Very highly/highly distributed nuclear receptors and moderately/scarcely expressed nuclear receptors regulate different signal pathways

A previous paper reported mouse nuclear receptor tissue expression profile using nucleic acid-binding-based RT-PCR technique [26, 27]. However, NR superfamily expression using more accurate DNA sequencing-based technique has not been profiled in human tissues. Comparing with that reported for mouse NR expression by Bookout et al. [26], our results on highly expressed NRs have the following features (Table 7): (1) our expression sequence tag (EST)-based data were more precise; (2) our data included 21 human tissues, but the previous report only examined mouse tissues; and (3) our data implicated that there are significant differences between human and mouse NR expressions, which had never been investigated before. Our data shows that humans have more NRs expressed in the central nerve system (CNS, 19 versus 11), metabolic system (40 versus 13), and cardiovascular system (19 versus 8). Therefore, our data of NR tissue expression profiles have provided valuable insight over potential NR functions in human tissues.

**Table 7:** Several findings in this study are significantly novel in comparing to what is published

Items	Expression profile of Nuclear receptors	
	Our findings	Cell paper (PMID: 16923397)
The number of nuclear receptors	48 known human NR	49 known mouse NR
Species	Human and mouse	Mouse
The number of tissues	21 human tissues and 17 mouse tissues	Only 39 mouse tissues
Analysis method	cDNA cloning and DNA sequencing experiments (EST database)	RT-PCR (high-throughput capacity)
Advantage of the method	More precise	–
NR groups based on their tissue distribution	–	Restricted (11), widespread (17), all tissues (21)
NR groups based on the expression level of nuclear receptors	Super high (12), high (5), low (3), super low activation (28)	–
Tissue groups based on number of highly expressed nuclear receptors	Super high (3/2 in human/mouse), high (4/5 in human/mouse), low (3/5 in human/mouse), supper low varieties (6/2 in human/mouse)	–
CNS (# human/mouse)	Brain, eye, nerve (19/1)	Eye, brainstem, cerebellum, cerebrum, corpus striatum, olfactory bulb, spinal cord, hypothalamus, and pituitary (11)
Gastroenteric system (# human/mouse)	Stomach, pancreas (5/5)	Tongue, stomach, duodenum, jejunum, ileum, colon, and gall bladder (13)
Metabolic system (# human/mouse)	Liver, kidney, adrenal gland, adipose, intestine, and muscle (40/14)	Liver, kidney, brown and white adipose, and muscle (13)
Immune system (# human/mouse)	Spleen and lymph node (4/6)	Spleen and thymus (2)
Cardiovascular system (# human/mouse)	Heart, lung, blood and trachea (19/6)	Aorta, heart, and lung (8)
Structural system (# human/mouse)	Skin (1/7)	Bone and skin (5)

**Fig. 2:**
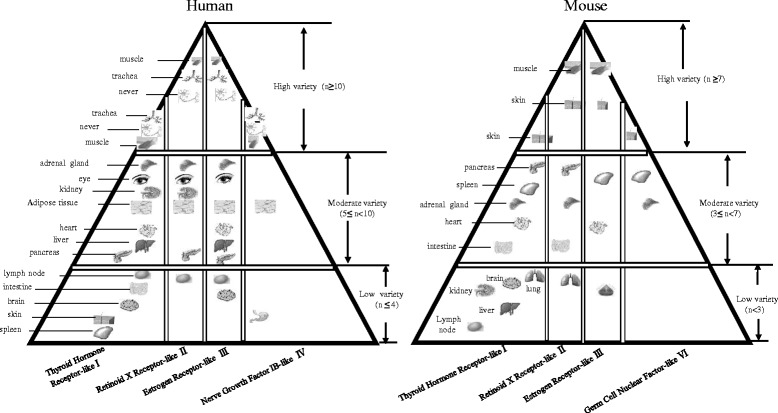
Our newly proposed “nuclear receptor pyramid” model in humans and mice constructed based on the number of variety of nuclear receptors expressed in tissues. *n*: the number of highly expressed nuclear receptors. Based on the numbers of NRs expressed in tissues, we classified tissues examined into three categories: high variety (expressed NRs *n* ≥ 10; *n* = numbers of the highly expressed NRs), moderate variety (expressed NRs 5 ≤ *n* < 10), and low variety (expressed 4 ≤ *n*) in a new nuclear receptor pyramid model in humans and high variety (expressed NRs *n* ≥ 7; *n* = numbers of the highly expressed NRs), moderate variety (expressed NRs 3 ≤ *n* < 7), and low variety (expressed NRs *n* < 3) in a new nuclear receptor pyramid model in mice

Based on the variety of NRs expressed in tissues, we classified tissues examined into three categories (Fig. 2), high variety (expressed NRs *n* ≥ 10; *n* = number of different types of highly expressed NRs), moderate variety (expressed NRs 5 ≤ *n* < 10), and low variety (expressed NRs *n* ≤ 4) in a new nuclear receptor pyramid model shown in Fig. 2 in humans. Similarly, we classified mouse NR pyramid model as high variety (expressed NRs *n* ≥ 7; *n* = numbers of the highly expressed NRs), moderate variety (expressed NRs 3 ≤ *n* < 7), and low variety (expressed NRs *n* < 3) (Fig. 2). These results suggested that the super high variety and moderate variety of NRs are found in tissues such as the muscle, trachea, and nerves in humans and in the muscle and skin in mice. Therefore, it can be concluded that these tissues may use NR pathways the most to regulate gene expression in response to developmental, physiological, and environmental stimulation. However, a high variety expression of NRs in the trachea has not been extensively reported [28]. It has been reported that NRs regulate skeletal muscle mitochondrial function [29] and the nervous system [30]. In addition, those tissues that have low variety of NRs may need fewer variety of NR pathways to regulate genes in response to developmental, physiological, and environmental stimuli; thus, they may also have other redundant pathways to carry out similar functions to that of NRs.

#### Correlation with oxygen sensors, angiogenic genes, and stem cell master regulators in human tissues

As shown in Table 6, NR functions in tissues may be involved in metabolism and stem cell-mediated tissue regeneration. However, it has been poorly characterized whether oxygen sensor genes such as prolyl hydroxylase domain-containing protein 2 (PHD2), hypoxia-inducible factor 1B (HIF1B), HIF1A, and HIF2A regulate NR expressions in tissues [31]. To determine the extents to which factors and NRs are related, we conducted correlation studies, with the hypothesis that if there is a positive functional correlation, the expression of the given factor (such as oxygen sensors, genes that regulate angiogenesis pathway, stem cell master genes, PRR, and inflammasome components) and the NR will increase or decrease together [1]. Similarly, we analyzed the correlation between NR expression and tissue methylation indices determined by the ratios between S-adenosyl methionine (SAM—the universal methyl donor)/S-adenosyl homocysteine (SAH—a methyltransferase inhibitor) and SAH levels [32].

**Fig. 3:**
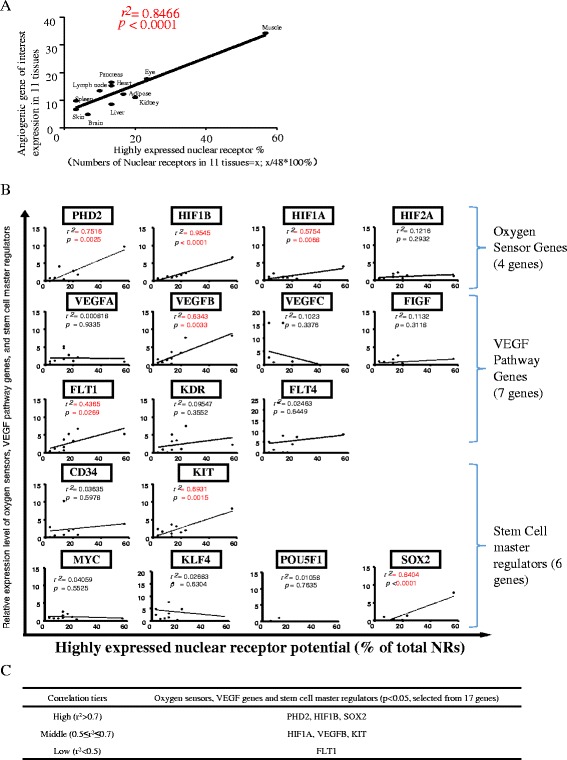
Oxygen sensors, VEGF pathway regulators, and stem cell master regulators may regulate nuclear receptor expression in human tissues (tissues: adipose, brain, eye, heart, kidney, liver, lymph node, muscle, pancreas, skin, spleen). **a** Highly expressed nuclear receptors in 11 tissues were strongly associated with angiogenic gene expression. **b** Correlation between highly expressed nuclear receptors and gene that regulate oxygen sensing, angiogenesis, and stem cells. **c** Correlation tiers between genes of interests and nuclear receptor expression in tissues. Abbreviations: PHD2: prolyl hydroxylase domain-containing protein 2; HIF1B: hypoxia-inducible factor-1 beta; HIF1/2A: hypoxia-inducible factor 1/2-alpha; VEGFA/B/C: vascular endothelial growth factor A/B/C; FIGF: C-fos-induced growth factor; FLT1/4: Fms related tyrosine kinase ¼; KDR: kinase insert domain receptor; MYC: MYC proto-oncogene; KIT: KIT proto-oncogene receptor tyrosine kinase; KLF4: Kruppel-like factor 4; POU5F1: POU class 5 homeobox 1; SOX2: SRY-box 2

As shown in Fig. 3a, b, we examined whether highly expressed NR potential (highly expressed NRs/total NRs × 100%) in tissues are correlated with tissue expression of four oxygen-sensing genes including PHD2, HIF1B, HIF1A, and HIF2A and seven vascular endothelial growth factor (VEGF) pathway genes including VEGFA, VEGFB, VEGFC, FIGF, FLT1, KDR, and FLT4, as well as six stem cell master genes including CD34, KIT, and four Yamanaka’s inducible pluripotent stem cell (IPSC) genes such as Myc, Kruppel-like factor 4 (KLF4), POU5F1 (octamer-binding transcription factor 4 (Oct4)), and sex determining region Y (SRY)-box 2 (Sox2) [33]. As shown in Fig. 3b, c, among 17 genes examined, the correlation of seven genes achieved statistical significance (*p* < 0.05). The highly expressed NR potentials were highly correlated with oxygen-sensing genes PHD2, HIF1B, and stem cell master regulator gene Sox2 (high correlation *r* ^2^ > 0.7). A moderate correlation was observed between highly expressed NRs and HIF1A, VEGFB, and KIT genes (0.5 ≤ *r* ^2^ ≤ 0.7). Low level correlation was observed between FLT1 and highly expressed NRs (*r* ^2^ < 0.5). These results suggested that the expression of oxygen-sensing genes PHD2, HIF1B, and HIF1A, VEGF pathway gene VEGFB and stem cell master gene SOX2, and KIT have a positive correlation with NR expression, and these genes may be either upstream regulators or downstream targets of NR signaling pathways.

#### Correlation with PRRs in human tissues

**Fig. 4:**
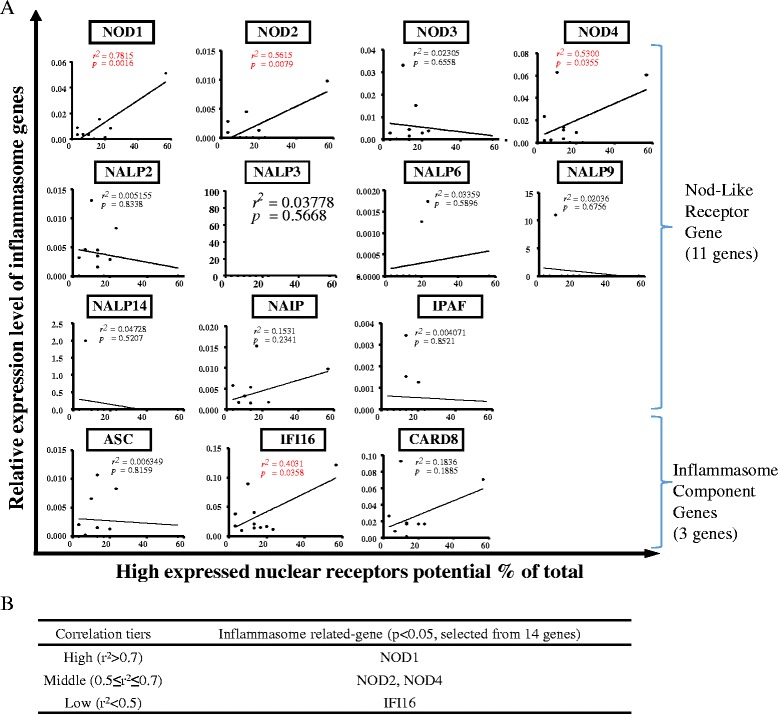
NLRs/inflammasome sensors may be either upstream regulators or downstream targets of nuclear receptors in human tissues (tissues: adipose, brain, eye, heart, kidney, liver, lymph node, muscle, pancreas, skin, spleen). **a** Correlation between inflammasome genes and highly expressed nuclear receptors. **b** Different correlation tiers show the level of statistically significant correlation between inflammasome genes and highly expressed nuclear receptors. Abbreviations: NOD1/2/3/4: nucleotide-binding oligomerization domain-like receptors 1/2/3/4; NALP2/3/6/9/14: NLR family pyrin domain containing 2/3/6/9/14; NAIP: NLR family apoptosis inhibitory protein; NLRC4: NLR family CARD domain containing 4; ASC: PYD and CARD domain containing; IFI16: interferon gamma-inducible protein 16; CARD8: caspase recruitment domain family member 8

Additionally, we addressed the question whether the highly expressed NRs have a positive correlation with the expression of PRR genes such as NLRs, AIM-2 (absent in melanoma-2), and IFI16 (interferon gamma-inducible protein 16) or genes of inflammasome components such as ASC (apoptosis speck-like CARD-containing protein) and CARD8 (caspase recruitment domain family member 8) [2, 8, 34]. As shown in Fig. 4, among 14 inflammasome-related genes examined, four PRR genes achieved statistically significant correlations (*p* < 0.05). The highly expressed NR potentials were highly correlated with microbial infection-sensing NOD1 [35] (high correlation *r* ^2^ > 0.7), moderately correlated with NOD2 and NOD4 (0.5 ≤ *r* ^2^ ≤ 0.7), and a weak correlation with nuclear DNA damage-sensing PRR IFI16 (*r* ^2^ < 0.5) [36].

It has been reported that nucleotide-binding oligomerization domain (NOD) proteins such as NOD1 and NOD2 are founding members of the NLR family, sense conserved motifs in bacterial peptidoglycan, and induce pro-inflammatory and anti-microbial responses [35]. It should be noted that three out of four PRRs, which include NOD1, NOD2, and NOD4 that are positively correlated with highly expressed NRs, activate inflammatory cascade independent of caspase-1 inflammasome complex. Nevertheless, IFI16, which is a PRR dominantly localized in the nucleus is a constituent of caspase-1 inflammasome complex, but it has a weak correlation with highly expressed NRs [37, 38]. Furthermore, NLRP3 [34], a PRR that is well identified as a component of caspase-1 inflammasome complex also failed to achieve a statistically significant correlation with highly expressed NRs. Therefore, this suggests that PRRs such as NOD1, NOD2, and NOD4 that function independently of caspase-1 are either upstream regulators or downstream targets of highly expressed NRs.

#### Correlation with methylation index in mouse tissues

DNA methylation has been recognized as one of the regulatory mechanisms underlying the expression of some NRs [39]. However, the question remains whether tissue methylation status regulates NR expression. There are two main intermediate compounds that determine the potential for methylation/demethylation in biological systems. S-adenosyl methionine (SAM) acts as a major methyl donor for many cellular methylation reactions of DNA, RNA, proteins, and lipids. In contrast, S-adenosyl homocysteine (SAH) is a potent inhibitor of biological transmethylation [40].

**Fig. 5:**
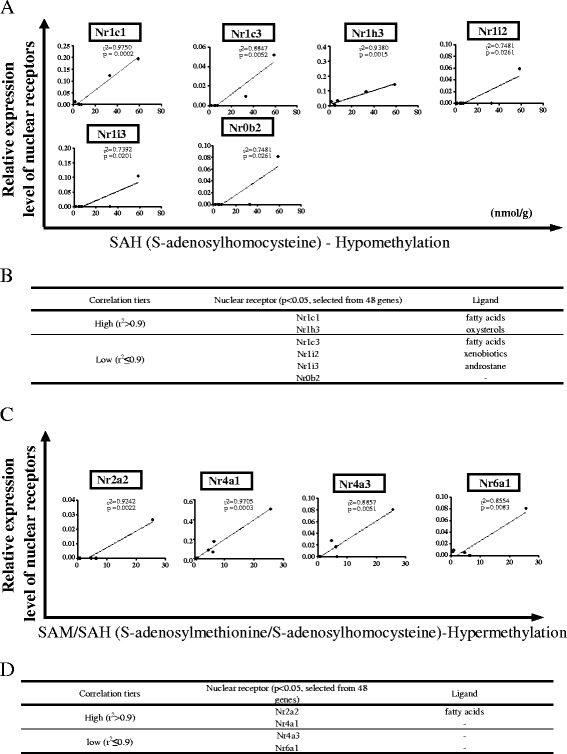
Tissue methylation status may determine the expression level of nuclear receptors in mouse tissues (tissues: mouse tissues: liver, brain, heart, kidney, lung, and spleen). **a** Correlation between nuclear receptors and hypomethylation status of the mouse tissues. **b** Different correlation tiers that depict the degree of association between hypomethylation status and nuclear receptor expression in mouse tissues. **c** Correlation between nuclear receptor expression and hypermethylation status of the mouse tissues. **d** Correlation tiers of hypermethylation status of the mouse tissues and nuclear receptor expression

To determine whether tissue methylation level determines NR expression, first we measured the tissue levels of SAH and SAM in six mouse tissues including the liver, brain, heart, kidney, lung, and spleen using liquid chromatography-electrospray ionization-tandem mass spectrometry [41]. We then analyzed the potential correlation between highly expressed NRs and the tissue hypomethylation determined by SAH (methyltransferase inhibitor) levels. Similarly, we examined whether a positive correlation exists between highly expressed NRs and tissue hypermethylation status determined by SAM/SAH (Fig. 5). Our data implicated that the NRs that undergo expression changes based on tissue methylation and demethylation status are mutually exclusive as we reported before [32].

As shown in Fig. 5a, among 48 NRs examined, 6 NRs showed a statistically significant positive correlation between NR expression and tissue hypomethylation status (*p* < 0.05). Two NRs including Nr1c1 (Pparα) and Nr1h3 were highly correlated with SAH levels in six tissues including the liver, brain, heart, kidney, lung, and spleen (high correlation *r* ^2^ > 0.9); four NRs including Nr1c3 (Pparγ), Nr1i2, Nr1i3, and Nr0b2 were moderately correlated with SAH levels (0.7 ≤ *r* ^2^ ≤ 0.9). Notably, most of the receptors that had increased expression levels in the presence of hypomethylation fall to class I NRs. Previously, it was shown that nutritional status can alter the methylation status of the PPARα gene and subsequently regulate its expression level both in rodent models and in humans [42]. It is highly likely that function of these receptors may also be increased during hypomethylation status as it provides easy access to these NRs to reach their response elements. Despite the observation that expression of certain class I NRs are increased in hypomethyation status, further experiments are needed to validate whether the function of these receptors are also enhanced.

In addition, as shown in Fig. 5c, d, among 48 NRs examined, 4 NRs achieved statistically significant correlation (*p* < 0.05) with tissue hypermethylation status. Two NRs including Nr2a2 and Nr4a1 were highly correlated with the SAM/SAH ratio in six tissues including the liver, brain, heart, kidney, lung, and spleen (high correlation *r* ^2^ > 0.9); two NRs including Nr4a3 and Nr6a1 show moderate correlation with the SAM/SAH ratio (0.8 ≤ *r* ^2^ ≤ 0.9). These results suggested that tissue hypermethylation status differentially regulates the tissue expression of NRs, and the tissue expression of five NRs may be significantly upregulated by hypermethylation. These results have demonstrated for the first time that tissue hypomethylation and hypermethylation status may have an impact on expression levels of two mutually exclusive groups of NRs [43].

We acknowledge that our data is not adequate to conclude that the NR expression changes that we observed are due to direct hypermethylation/hypomethylation of the particular NR gene. Tissue methylation status may regulate NR expression indirectly via other mediators. Though we did not observe expression changes on estrogen receptor-alpha (ER-α) in our mouse dataset, previously it was shown that methylation status of the ER-α gene determines its expression in the colon, blood, lung, heart, prostrate, and ovary in humans [44–48]. This was extensively studied in human breast cancer cell lines, where loss of ER expression and acquired hormone resistance was attributed to hypermethylation of the ER gene [44, 49]. Therefore, to conclude whether the NR expression changes we observed are due to direct methylation of the particular genes requires further experimental validation. Furthermore, pathophysiological relevance of the methylation status of the tissues and NR expression need to be tested in the future. Also, it is possible that upregulation of certain NRs may regulate the tissue methylation status via unknown pathways.

It should be noted that the values that determined the correlation tiers in Fig. 5 are different from those of Figs. 3 and 4. This is because the basal levels were different as Fig. 5 demonstrates the data obtained from mice and Figs. 3 and 4 depict the data obtained from human tissues. Also, when analyzing the correlation, NR potential was taken into account in Figs. 3 and 4, while the correlation was calculated for each and individual NR in Fig. 5.

### Nuclear receptor sequence changes and mutations are associated with increased risk for development of metabolic, cardiovascular, and autoimmune diseases, hormone insensitivity/resistance, and cancers

Genome-wide association studies (GWASs) have investigated potential genetic factors that explain inter-individual variations in response to NR ligand stimulations in various pathologies [50]. Given that susceptibility to complex human metabolic diseases is likely a result of genes operating as part of functional modules rather than individual effects, association analysis methods hold promise in discovering additional associations from existing GWAS data [51]. Previous GWAS studies have been reported for NRs in some diseases such as liver injury [50], osteoporosis, sarcopenia, and obesity. However, it is unclear whether the GWAS data on NRs are associated with globally increased genetic risks for metabolic diseases and autoimmune disease, such as rheumatoid arthritis, obesity, diabetes, and vascular atherosclerosis in human populations.

To address this issue, we examined the GWAS database (http://www.gwascentral.org/) for all the NRs. As shown in Tables 8 and 9, 45 out of 48 NRs with sequence changes or mutations were associated with rheumatoid arthritis, obesity, diabetes, and vascular disease and atherosclerosis. In addition, two NRs such as PPARA and NR3C2 variations were associated with certain lipid metabolite traits (Table 8). Despite the fact that AR exerts pro-inflammatory effects like PPARD and RXRA, it was much less associated with development of obesity and diabetes unlike PPARD and RXRA (Table 8). Finally, NR2F2 variations were not associated with the diseases examined except in one diabetes study.

**Table 8:** 45 out of 48 nuclear receptors with sequence changes or mutations are associated with increased risks of human rheumatoid arthritis, obese, diabetes, and metabolic vascular diseases

Gene	Diseases																						
	Rheumatoid arthritis		Obese			Diabetes												Vascular dis. and atherosclerosis					
	Phenotype ID (HGVPM)		Phenotype ID (HGVPM)			Phenotype ID (HGVPM)												Phenotype ID (HGVPM)					
	38	235	564	1111	1114	4	5	74	822	825	826	827	569	769	1081	1538	3639	563	568	3602	3611	3640	3616
THRA	*	*	*	*	*	*	*		*	*	*	*	*	*	*			*	*				
THRB	*	*	*	*	*	*	*	*	*	*	*	*	*	*	*	*		*	*				
RARA	*		*	*	*	*			*	*	*	*	*	*	*	*		*	*				
RARB	*		*	*					*	*	*	*	*		*	*							
RARG				*	*				*	*	*	*		*	*	*							
PPARA	*	*	*	*	*	*	*		*	*	*	*	*	*	*	*		*	*	*	*	*	
**PPARD**	*	*	*	*	*	*	*		*	*	*	*	*	*	*	*		*	*				
PPARG			*	*	*		*		*	*	*	*	*	*	*	*		*	*				
NR1D1			*	*	*				*	*	*	*	*		*	*		*	*				
NR1D2	*	*	*	*	*	*	*		*	*	*	*	*	*	*	*		*	*				
RORA	*	*	*	*	*	*	*		*	*	*	*	*	*	*	*	*	*	*				
RORB	*	*	*	*	*	*	*		*	*	*	*	*	*	*	*		*	*				
RORC	*	*	*	*	*	*			*	*	*	*	*	*	*	*		*	*				
NR1H3	*	*	*	*	*	*	*		*	*	*	*	*	*	*	*		*	*				
NR1H2				*	*				*	*	*	*			*	*							
NR1H4	*	*	*	*		*	*		*	*	*	*	*	*	*	*		*	*				
VDR	*	*	*	*					*	*	*	*	*		*	*							
NR1I2	*	*	*	*		*	*		*	*	*	*	*	*	*	*		*	*				
NR1I3	*	*	*	*	*	*			*	*	*	*	*	*	*	*		*	*				
HNF4A	*	*	*	*					*	*	*	*	*		*	*			*				
HNF4G	*	*	*	*	*	*	*		*	*	*	*	*	*	*	*		*	*				
**RXRA**	*	*	*	*		*	*		*	*	*	*	*	*	*	*		*	*				
RXRG	*	*	*	*	*	*	*		*	*	*	*	*	*	*	*		*	*				
NR2C1	*	*	*	*	*	*	*		*	*	*	*	*	*	*	*		*	*				
NR2C2	*	*	*	*	*	*	*		*	*	*	*	*	*	*	*		*	*				
NR2E1	*	*	*	*	*	*	*		*	*	*	*	*	*	*	*		*	*				
NR2E3				*	*				*	*	*	*		*	*	*							
NR2F1	*	*	*	*	*	*			*	*	*	*	*	*	*	*		*	*				
NR2F2							*																
NR2F6				*	*				*	*	*	*		*	*	*							
ESR1	*	*	*	*	*	*	*		*	*	*	*	*	*	*	*		*	*				
ESR2	*	*	*	*	*	*	*		*	*	*	*	*	*	*	*		*	*				
ESRRA					*				*	*	*	*		*	*								
ESRRB	*	*	*	*	*	*	*		*	*	*	*	*	*	*	*		*	*				
ESRRG	*	*	*	*	*	*	*		*	*	*	*	*	*	*	*		*	*				
NR3C1	*	*	*	*	*	*	*		*	*	*	*	*	*	*	*		*	*				
NR3C2	*	*	*	*	*	*	*		*	*	*	*	*	*	*	*		*	*	*	*	*	*
PGR	*	*	*	*	*	*	*		*	*	*	*	*	*	*	*		*	*				
**AR**	*	*	*				*						*					*	*				
NR4A1	*	*	*	*	*	*	*		*	*	*	*	*	*	*	*		*	*				
NR4A2	*	*	*	*	*	*	*		*	*	*	*	*	*	*	*		*	*				
NR4A3	*	*	*	*	*	*	*		*	*	*	*	*	*	*	*		*	*				
NR5A1	*	*	*	*	*	*			*	*	*	*	*	*	*	*		*	*				
NR5A2	*	*	*	*	*	*	*		*	*	*	*	*	*	*	*		*	*				
NR6A1	*	*	*	*	*	*	*		*	*	*	*	*	*	*	*		*	*				

Nuclear receptors marked with bold have a pro-inflammatory role

**Table 9:** Study ID and phenotype ID from Table 8

Disease	Study ID (HGVST)	Study name (GWAS)	Phenotype ID (HGVPM)	Phenotype property	Title (phenotype HGVPM)
Rheumatoid arthritis	27	Rheumatoid arthritis	38	Anti-cyclic citrullinated peptide-positive rheumatoid arthritis	38: Stage 1 anti-CCP-positive rheumatoid arthritis status
	185	Rheumatoid arthritis in the Spanish population	235	Rheumatoid arthritis	235: Rheumatoid arthritis
Obese	640	Body mass index	1111	Body mass index	1111: Phenotype method for body mass index
	308	Adult body mass index in a British population	564	Body mass index	564: Adult body mass index measurement
	641	Meta-analysis of 32 genome-wide association studies for waist-hip ratio adjusted for body mass index	1114	Waist-hip ratio	1114: Phenotype method for waist-hip ratio
Diabetes	463	Glycemic traits	825	Fasting glucose-related: fasting plasma glucose	825: Phenotype method for fasting glucose-related: fasting plasma glucose
	463	Glycemic traits	827	Fasting glucose-related: homeostatic model assessment of beta-cell function	827: Phenotype method for fasting glucose-related: homeostatic model assessment of beta-cell function
	463	Glycemic traits	822	Fasting insulin-related: fasting insulin	822: Phenotype method for fasting insulin-related: fasting insulin
	463	Glycemic traits	826	Fasting insulin-related: homeostatic model assessment of insulin resistance	826: Phenotype method for fasting insulin-related: homeostatic model assessment of insulin resistance
	618	Glycated hemoglobin levels	1081	Glycated hemoglobin levels	1081: Phenotype method for glycated hemoglobin levels
	313	Log10 glycosylated hemoglobin in a British population	569	Log10 glycosylated hemoglobin	569: Log10 glycosylated hemoglobin measurement
	433	Glucose levels 2 h after an oral glucose challenge	769	2-h glucose challenge	769: Phenotype method for 2-h glucose challenge
	5	Type II diabetes mellitus	4	Type II diabetes	4: T2D status
	3	Type II diabetes mellitus	5	Type II diabetes	5: T2D status
	52	Type II diabetes mellitus in American Indians	74	Early onset type II diabetes mellitus	74: Phenotype method forearly onset type II diabetes mellitus
	907	Proinsulin levels	1538	Proinsulin levels	1538: Phenotype method for proinsulin levels
	1827	Metabolite quantitative traits	3639	a-Glucose	3639: Phenotype method for a-glucose
Metabolic vascular disease	312	Serum cholesterol levels in a British population	568	Serum cholesterol	568: Serum cholesterol measurement
	1827	Metabolite quantitative traits	3602	Lipids (CH3)	3602: Phenotype method for Lipids (CH3)
	1827	Metabolite quantitative traits	3611	Lipids (CH2)	3611: Phenotype method for Lipids (CH2)
	1827	Metabolite quantitative traits	3616	Lipids (CH2CO)	3616: Phenotype method for Lipids (CH2CO)
	1827	Metabolite quantitative traits	3640	Lipids (CH=CH*CH2CH2)	3640: Phenotype method for Lipids (CH=CH*CH2CH2)
	307	Systolic blood pressure in a British population	563	Systolic blood pressure	563: Systolic blood pressure measurement

These results suggest that NRs may be very important factors in determining the susceptibility and progression of metabolic disorders including obesity, diabetes, and atherosclerosis. Also, our GWAS analysis suggests that NRs may play an important role in the progression of autoimmune disorders such as rheumatoid arthritis. Most interestingly, the NR mutations associated with various metabolic disorders and autoimmune diseases are different. This observation can be supported by multiple publications that had demonstrated NRs play an important role in immune cells. Especially, the PPARs are highly expressed in human CD4+ T cells [52], and the role of PPAR agonists in the treatment of autoimmune disorders had been extensively discussed [53, 54]. It was shown that activation of T cells was dramatically decreased in the presence of PPARA and PPARG agonists and suppressed pro-inflammatory cytokine secretion [52]. In addition to PPARs, other NRs such as ROR-γt were found to regulate differentiation of CD4+ T helper 17 (Th17) subset [55]. However, RAR/RXR dimerization exerts contrasting effects to that of ROR-γt by enhancing Foxp3 transcription factor positive inducible T-regulatory cells (Tregs) while inhibiting Th17 differentiation [56]. Therefore, it is evident that the cross talk between NRs play a critical role in the immunity and development of autoimmune disorders.

In addition to the GWAS analysis, we further performed the cause-effect analysis using the mouse genome informatics (MGI) database (www.mousemine.org) that contains a comprehensive compilation of genomic and phenotypic data from NR transgenic and gene knockout mouse models. In Table 10, 26 NR deficiencies lead to four groups of abnormalities, including (1) hormone insufficiency/insensitivity/resistance, (2) cancers, (3) autoimmune/immunodeficiency diseases, and (4) metabolic cardiovascular diseases. The NRs in the hormone insufficiency/insensitivity/resistance group include NRs THRA, THRB, RARB, NR1H3, VDR, NR2E3, NR2F1, NR2F2, ESR1, ESRRB, NR3C1, NR3C2, PGR, AR, NR4A2, NR4A3, NR5A1, and NR0B1. The NRs in the cancer group include RARA (acute promyelocytic leukemia), NR1H4 (hepatocellular carcinoma), ESR1 (breast cancer), and AR (prostate cancer). The NRs in the autoimmune/immunodeficiency disease group include PPARG (systemic lupus erythematosus), RORC (immunodeficiency), and RXRA (systemic lupus erythematosus). The NRs in the metabolic cardiovascular diseases group include RARA (cardiomyopathy), PPARD (diabetes mellitus), PPARG (diabetes mellitus, lipodystrophy, obesity, pulmonary hypertension), HNF4A (diabetes mellitus), NR2F2 (congenital heart defects), ESR1 (myocardial infarction), NR3C2 (hypertension), AR (obesity), and NR0B2 (obesity). Taken together, the results of the GWAS association studies and MGI casual analysis suggest that NRs play significant roles in maintaining the homeostasis and suppressing various hormonal diseases, cancers, autoimmune diseases/immunodeficiency, and metabolic cardiovascular diseases.

**Table 10:**
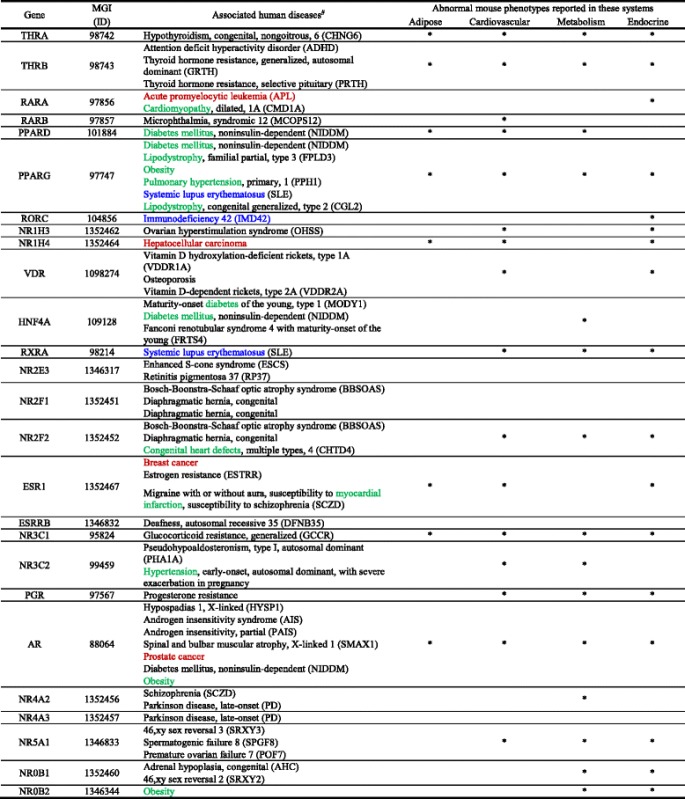
The Mouse Genome Informatics (MGI) database shows that 26 nuclear receptor deficiencies lead to abnormal metabolism and endocrine and cardiovascular phenotypes in mice

^#^Disease association of human genes are from the NCBI mim2gene_medgen file and include annotations from OMIM, NCBI curation, Gene. *Abnormal mouse phenotypes. Of note, red fonts indicate cancers, blue fonts indicate autoimmune diseases, and green fonts indicate metabolic disorders such as obesity

To further determine whether many NRs serve as homeostasis-associated molecular pattern receptors (HAMPRs) by inhibiting inflammation, we conducted an extensive literature survey to find out experimentally validated data to prove our hypothesis. As shown in Table 11, the level of 10 hormone ligands of NRs was changed with inflammatory diseases. The ligands of class-I thyroid hormone receptor-like group including vitamin A, fatty acids, and prostaglandins levels were reduced in the presence of inflammatory disorders, suggesting that they have the potential to exert anti-inflammatory effects. In addition, retinoids and estrogen inhibited inflammatory intestinal disease and atherosclerosis respectively. Moreover, testosterone suppressed Crohn’s disease.

**Table 11:** Hormone ligand level changes are associated with inflammatory diseases

NRNC symbol	Ligand(s)	Inflammatory disease	Ligand level change	PMID
Class I—thyroid hormone receptor-like				
NR1A1	Thyroid hormone	Inflammatory bowel diseases	↑	8562993
NR1A2				
NR1B1	Vitamin A	Chronic obstructive pulmonary disease	↓	26339144
NR1B2				
NR1B3				
NR1C1	Fatty acids, prostaglandins	Inflammatory bowel disease	↓	27631140
NR1C2				
NR1C3				
NR1F1	Cholesterol	Atherosclerotic cardiovascular disease	↑	21686232
NR1F2				
NR1F3				
NR1H3	Oxysterols	Inflammatory bowel diseases	↑	24024145
NR1H2				
NR1H4				
Class II—retinoid X receptor-like				
NR2B1	Retinoids	Inflammatory intestinal disease	↓	23690441
NR2B2				
NR2B3				
Class III—estrogen receptor-like				
NR3A1	Estrogens	Atherosclerosis	↓	12816884
NR3C1	Cortisol	Obesity	↑	12466357
NR3C2	Aldosterone	Renal fibrosis	↑	26730742
NR3C4	Testosterone	Crohn’s disease	↓	26020563

Finally, we also searched for the evidence in the literature where gene knockout and activation approaches of NRs were used to determine the pathological phenotypes. Twelve out of 15 NRs including NR1A1, NR1C3, NR1D1, NR1H3, NR1H2, NR1H4, NR2F2, NR3A1, NR3B2, NR4A1, NR4A3, and NR0B2 have anti-inflammatory roles as shown in Table 12. The three NRs NR1C2, NR2B1, and NR3C4 did not show any anti-inflammatory properties. Taken together, these results suggested that most human and mouse NRs have anti-inflammatory functions in various tissues and cell types.

**Table 12:** 12 out of 15 nuclear receptors have anti-inflammatory roles reported in the literature

Gene name (full name)	NRNC symbol	Tissue/cell type	Purpose	Treat	Suppressed	Induced	PMID	Inflam.
					Cytokines/signaling			
Class I—thyroid hormone receptor-like								
THRA	NR1A1	Aorta macrophages	Atherosclerosis	KO	–	IL-1β, NFκB, TNF-α	24797634	Anti
PPARG	NR1C3	Mouse cancer model	Tumor growth and angiogenesis	Act	IL-17	–	23619236	Anti
NR1D1	NR1D1	Peritoneal macrophages	Aging- or obesity-associated impairment of clockwork and inflammation	Act	Ccl2, ERK, p38	–	24307731	Anti
		Mice macrophages	Circadian clockwork and inflammatory disease	KO	–	IL-6	22184247	
NR1H3	NR1H3	Mice plasma and kidney	Normal and diabetic kidney	KO	–	Nox2, Ncf1, MDA, TLR2, ICAM1, IL-1β, CD68	24201575	Anti
NR1H2	NR1H2	Mice plasma and kidney	Normal and diabetic kidney	KO	–	Nox2, Ncf1, TLR2, ICAM1, IL-1β, CD69, MDA(urinary)	24201575	Anti
		Macrophage cell line	LPS treat	Act	TNF-α, IL-1β, IL-6, IL-12p40	–	23099324	
		ob/ob mouse liver	Cellular lipid metabolism	Block	–	Cox-2, MCP-1, MIP-2	24206663	
NR1H4	NR1H4	Obese mice liver	Obesity-related metabolite disorder	Ace	–	Mmp13, Cxcl2, Cxcl8, Cxcl14, IL-1β, IL-6, TNF-α	25425577	Anti
PPARD	NR1C2	Epithelial cells		Act	–	COX-2	24763687	Pro
Class II—retinoid X receptor-like								
RXRA	NR2B1	Spleen macrophages	Age-related disease	Act	–	COX-2, NF-kB, IL-6	24051096	Pro
NR2F2	NR2F2	Prostate cancer	Prostate cancer	Act	TGF-β	–	23201680	Anti
Class III—estrogen receptor-like								
ESR1	NR3A1	Male mice	Obesity	KO	IL-10	IL-1β, TNF-α, IL-6	25373903	Anti
		Astrocytes	Neuroprotective	Act	CCL2, CCL7	–	23804112	
ESR3	NR3B2	Mice	Intestine tumor	KO	–	TGF-β	24104551	Anti
AR	NR3C4	Prostate cancer cells	Prostate tumorigenesis	KD	–	AKT	25527506	Pro
		Hepatocellular carcinoma cells	Cell adhesion and migration	KO	–	PI3K/AKT	24944078	
Class IV—nerve growth factor IB-like								
NR4A1	NR4A1	Macrophages	Atherosclerotic lesions	KO	–	IL-4	23288947	Anti
		Bone marrow-derived macrophages (BMM)	Atherosclerotic lesions	KO	–	IL-12, IFN-δ, SDF-1α	22194623	
		Macrophage	Atherosclerotic lesions	KO	–	TNF-α, TLR-4, NFκB	22194622	
NR4A3	NR4A3	Mast cells	Vascular biology and inflammation	KO	–	IL-13, MCP-1, TNF-α	24586680	Anti
		Hematopoietic stem cells	Atherosclerotic lesions	KO	–	Ly6C(+) monocytes	24806827	
		Endothelial cells	Atherosclerotic lesions	KO	–	VCAM-1, ICAM-1	20558821	
Class O—miscellaneous								
NR0B2	NR0B2	Mice kidney	Inflammasome	KO	–	IL-1β, IL-18, NLRP3, ASC	25655831	Anti

Abbreviations: *KO* knockout, *Act* activation, *Ace* acetylation, *Cxcl* Cxc ligand, *IL* interleukin, *MCP* monocyte chemotactic protein, *Mmp* matrix metallopeptidase, *TLR* Toll-like receptor, *VCAM* vascular cell adhesion molecule, *ICAM* intercellular adhesion molecule, *Inflam* inflammation, *Anti* anti-inflammatory, *Pro* pro-inflammatory

### Nuclear receptors have the tendency to be downregulated than being upregulated in autoimmune and metabolic diseases and cancers

In order to determine the overall roles of NRs in modulating the pathogenesis of human autoimmune diseases, metabolic diseases, and cancer, we examined the expression changes of 48 NRs in eight human diseases using the microarray datasets (https://www.ncbi.nlm.nih.gov/gds/) deposited by other investigators in the NIH-GEO dataset database. The microarray datasets we analyzed were conducted on various pathological settings including autoimmune disease rheumatoid arthritis, and five metabolic diseases such as familial hypercholesterolemia, type 2 diabetes, type 1 diabetes, obesity, hyperhomocysteinemia, and also hypertension. We analyzed The Cancer Genome Atlas (TCGA) database to determine NR expression changes in human cancers.

As shown in Table 13 (A), three NRs were upregulated but nine NRs were downregulated in the synovial tissue of patients with rheumatoid arthritis. Similarly, in Table 13 (B), 7 NRs were upregulated and 11 NRs were downregulated in T cells from patients with familial hypercholesterolemia. Also, we analyzed the monocytes isolated from patients with familial hypercholesterolemia, peripheral blood from patients with metabolic syndrome, arterial tissue from patients with type 2 diabetes, peripheral blood mononuclear cells from patients with type 1 diabetes, adipose stem cells and omental adipose tissue from morbidly obese patients, aortic smooth muscle cells from patients with hyperhomocysteinemia, and carotid artery atheromatous plaques from patients with hypertension. The results showed that NRs have the tendency to be downregulated during metabolic disorders and autoimmune disorders rather than being upregulated. However, this trend was not observed in morbidly obese patients where equal numbers of NRs were upregulated and downregulated (Table 13 (B)). To further consolidate the finding, we analyzed the NR expression changes in the presence of proatherogenic stimulus oxidized low-density lipoprotein (Ox-LDL) in human aortic endothelial cells (HAECs). This analysis also showed that NRs tend to be downregulated than upregulated with prolonged Ox-LDL treatment (Table 13 (C)). Taken together, these results suggested that NRs have the tendency to be downregulated than upregulated during human autoimmune rheumatoid arthritis and metabolic diseases, and this tendency of NRs was more obvious in autoimmune arthritis than in metabolic diseases.

**Table 13:** Nuclear receptors are more downregulated than upregulated in human diseases

Disease	Tissue/cell type	Number		Upregulated gene		Downregulated gene		PMID/GEO ID
		Up	Down					
A. Nuclear receptors have the tendency to be downregulated than being upregulated in rheumatoid arthritis								
					Fold*		Fold*	
					∆		∆	
Rheumatoid arthritis	Synovial tissue	3	9	NR1H3	2.08	NR1A1	−20	24690414/GSE55235
				NR1I1	2.12	NR1C3	−2.56	
				NR3A1	2.59	NR1D1	−20	
						NR2F1	−2.94	
						NR3C3	−2	
						NR3C4	−2.22	
						NR4A1	−4.76	
						NR4A2	−10	
						NR4A3	−3.85	
B. Nuclear receptors are more downregulated than upregulated in metabolic diseases in humans								
					Fold**		Fold**	
					∆		∆	
Family hypercholesterolemia	T cells	7	11	NR1B3	1.41	NR1A1	−1.3	–/GSE6088
				NR1C3	1.94	NR1B1	−1.22	
				NR1I1	1.75	NR1B2	−1.72	
				NR3C4	1.97	NR1C1	−1.72	
				NR4A2	2.03	NR1F1	−2	
				NR4A3	1.5	NR1F2	−1.79	
				NR0B1	2.36	NR1H3	−1.28	
						NR3B3	−2.08	
						NR3C1	−1.22	
Family hypercholesterolemia	Monocytes	10	12	NR1B1	1.23	NR1A1	−2.27	19040724/GSE6054
				NR1F3	2.38	NR1B2	−1.72	
				NR1I1	1.31	NR1C1	−1.59	
				NR2F6	2.61	NR1F1	−1.52	
				NR3B2	1.33	NR1H3	−1.28	
				NR4A1	1.94	NR2A2	−2.13	
				NR4A2	2.06	NR2C1	−1.47	
				NR5A1	2.1	NR2C2	−1.19	
				NR6A1	1.62	NR2E3	−2.63	
				NR0B1	2.02	NR3A1	−2	
						NR3A2	−1.79	
						NR3C1	−1.28	
Metabolic syndrome	Peripheral blood	0	1			NR4A3	−1.54	21368773/GSE23561
Type 2 diabetes	Arterial tissue	0	2			NR1B2	−1.28	22340758/GSE13760
						NR3C3	−1.12	
Type 1 diabetes	Peripheral blood mononuclear cell	1	2	NR3C4	1.2	NR1F1	−1.89	–/GSE55100
						NR4A3	−1.2	
Morbidly obese	Adipose stem cells	3	3	NR4A1	8.06	NR1A1	−1.2	24040759/GSE48964
				NR4A2	12.64	NR1D1	−1.2	
				NR4A3	4.44	NR2C2	−1.25	
Morbidly obese	Human omental adipose tissue	1	1	NR4A2	3.5	NR2B3	−2.44	20678967/GSE15773
Homocysteine (100 μM)	Human aortic smooth muscle cells	3	3	NR2B3	1.2	NR1H3	−1.33	18602108/GSE9490
				NR3A2	1.61	NR2F2	−1.7	
				NR4A3	1.49	NR4A2	−1.24	
Hypertension	Carotid artery atheromatous plaques	0	2			NR1A2	−2.04	23660665/GSE43292
						NR3C3	−2	
C. Nuclear receptors are significantly downregulated than upregulated in human aortic endothelial cells (HAECs) treated with oxidized low-density lipoproteins (Ox-LDLs) in a time-dependent manner								
					Fold*		Fold*	
					∆		∆	
Treated with Ox-LDL for 6 h	HAEC	4	3	NR1I2	2.18	NR1B2	−3.57	19279231/GSE13139
				NR2A2	3.8	NR1F1	−5.26	
				NR3A2	3.04	NR4A1	−2.56	
				NR5A2	4			
Treated with Ox-LDL for 12 h		0	7			NR1B1	−2.86	
						NR1B2	−5.56	
						NR1F1	−2.86	
						NR1H4	−5.88	
						NR2A2	−2.04	
						NR3A1	−2.94	
						NR3C3	−2.33	
Treated with Ox-LDL for 24 h		5	7	NR1C1	2.24	NR1B1	−2.56	
				NR1I1	2.01	NR1B2	−2.17	
				NR3A2	5.09	NR1B3	−2.04	
				NR3B2	2.34	NR1H4	−4.55	
				NR5A2	5.81	NR1I2	−2.5	
						NR2A2	−2.13	
						NR2F6	−4.55	

Abbreviations: *HAECs* human aortic endothelial cells, *Ox-LDL* oxidized low-density lipoprotein

*Fold change > 2

**Fold change > 1.2

Specifically, our data shows that NR1C1 (PPARα) is among the downregulated genes in familial hypercholesterolemia. NR1C1 is one of the primary modulators in fatty acid oxidation and apolipoprotein synthesis [23]. This receptor was also found abundantly in the vascular wall and in human macrophages and was shown to exert anti-inflammatory and anti-atherogenic effects [57]. Therefore, downregulation of this gene may contribute to hypercholesterolemia and also to progression of atherosclerotic events. PPARα agonists are widely used to correct hyperlipidemia and were shown to reduce mortality and morbidity due to cardiovascular events [58]. Furthermore, we observed that NR1C3 (PPARγ) is downregulated in patients with rheumatoid arthritis. Previously, PPARγ was reported to have a negative effect on oxidative stress, and therefore, it was suggested that concomitant use of PPARγ agonists with other treatments will give additional therapeutic benefits against rheumatoid arthritis [59].

NRs play an important role in the development and progression of cancers. For an example, the roles of androgen receptors in breast and prostate cancers are well documented [60–62]. We analyzed the NR expression in 17 different types of cancers in TCGA database. Similar to the observation we elaborated above, our data revealed that the tendency of NRs to be downregulated is more than being upregulated. NR1H2 receptor was downregulated in as many as seven types of cancers, NR2B2 in six types, and NR1B1 and NR1A1 in five types of cancers (Table 14). However, specifically NR1A1 (RAR α) and also NR1B1 (RAR β) are associated with progression of estrogen-dependent breast cancers [63]. This is contrasting to our observation of the expression of these two receptors in other types of cancers. Nevertheless, activation of NR1H2 which falls in to liver X receptors was shown to inhibit proliferation of HT29 colorectal cancer cells [64]. Therefore, this suggests that NR1H2 can be a potential therapeutic target for the treatment of many types of cancers.

**Table 14:**
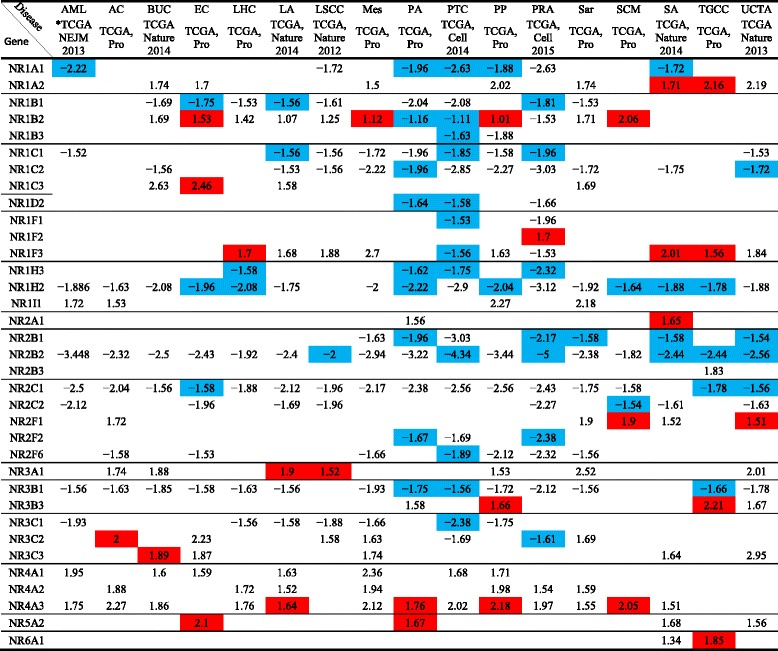
More nuclear receptors are downregulated in 17 different types of human cancers

The numbers in the cells represent fold change (≥ 1.5); positive symbol means upregulation while negative symbol means downregulation. Red color means significant upregulation while blue color means significant downregulation (*p* < 0.05). No color means no significance (*p* ≥ 0.05), and blank means no data is available

Abbreviations: *TCGA* The Cancer Genome Atlas, *Pro* provisional, *AML* acute myeloid leukemia, *AC* adrenocortical carcinoma, *BUC* bladder urothelial carcinoma, *EC* esophageal carcinoma, *LHC* liver hepatocellular carcinoma, *LA* lung adenocarcinoma, *LSCC* lung squamous cell carcinoma, *Mes* mesothelioma, *PA* pancreatic adenocarcinoma, *PTC* papillary thyroid carcinoma, *PP* pheochromocytoma and paraganglioma, *PRA* prostate adenocarcinoma, *Sar* sarcoma, *SCM* skin cutaneous melanoma, *SA* stomach adenocarcinoma, *TGCC* testicular germ cell cancer, *UCEC* Uterine Corpus Endometrial Carcinoma

*Reference

**Fig. 6:**
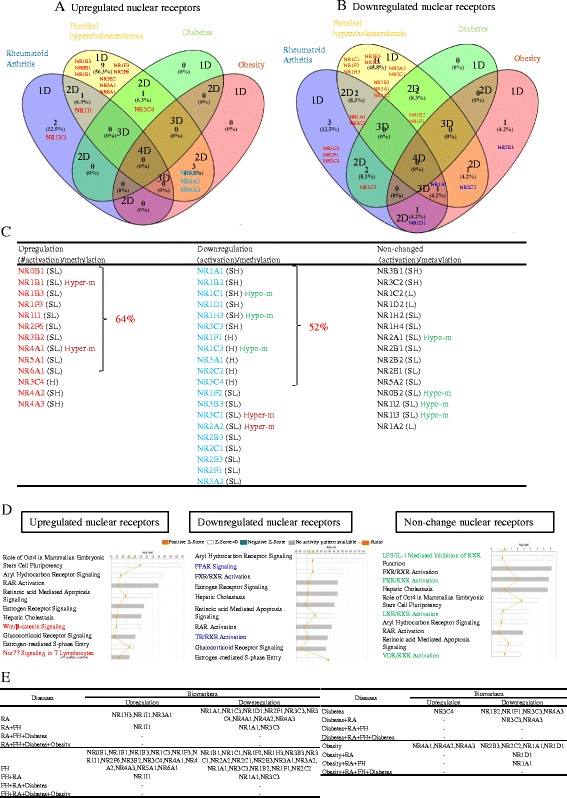
Venn analysis of significantly changed nuclear receptor expression among four different tissues. **a**, **b** Venn diagram shows the number of significantly upregulated and downregulated nuclear receptors in four different pathologies respectively (blue, yellow, green and red represent rheumatoid arthritis/ familial hypercholesterolemia/diabetes/obesity). **c** The nuclear receptor genes that are upregulated, downregulated, and without any expression changes in four pathologies of interest. **d** The signaling pathways that are regulated by nuclear receptor genes that are upregulated, downregulated, and have no expression changes in four pathologies of interest. **e** A list of nuclear receptors that can be used as biomarkers to detect indicated pathologies

To determine the features of those human diseases-modulated NRs, we performed Venn analysis as we previously reported [15]. The Venn analysis/diagram is a very useful analytical tool as it helps to clearly visualize the NRs that are shared between the different diseases analyzed. In Fig. 6a, b, the results show that NR expression changes in human diseases are not shared. In four human diseases analyzed by the Venn analysis, 13 NRs were upregulated, 20 NRs were downregulated, and 15 NRs were not changed in their expression levels (Fig. 6c). Of note, 10 out of 13 upregulated NRs in human diseases were from the scarcely distributed group shown in Table 6, 11 out of 20 NRs downregulated in human diseases were from the very highly distributed and highly distributed groups in Table 6, and 13 out of 15 NRs whose expressions were not changed in human diseases were from the moderately distributed and scarcely distributed groups shown in Table 6. Notably, the tissue expression of 3 NRs out of 20 disease-mediated downregulated NRs including NR1C1, NR1H3, and NR1C3 were correlated with tissue hypomethylated index SAH levels (Fig. 5b), 4 out of 15 NRs whose expressions were not changed were correlated with hypomethylated index SAH levels, and none of the NRs in the disease-upregulated group were correlated with hypomethylated index SAH levels (Fig. 6c). These findings are in a good correlation with tissue hypomethylation function in promoting inflammation as we reported [32, 65], suggesting that hypomethylation-promoting hyperhomocysteinemia may facilitate inflammation via inhibiting the expression of those human disease-downregulated NRs and also keep the stable expression of those non-disease-changed NRs.

In order to determine functional significances of disease-modulated NR expression, we analyzed the top 10 signaling pathways with the disease-upregulated NRs, disease-downregulated NRs, and non-disease-modulated NRs using the Ingenuity Pathway Analyzer (https://www.qiagenbioinformatics.com/products/ingenuity-pathway-analysis/). As shown in Fig. 6d; in addition to the shared pathways among three groups of NRs, two pathways, Wnt/β-catenin signaling, and Nur77 signaling were specifically associated with the disease-upregulated NRs; two other pathways such as peroxisome proliferator-activated receptor (PPAR) signaling and thyroid hormone receptor (TR)/retinoid X receptor (RXR) activation were specifically associated with the downregulated NRs; and four pathways including LPS/IL-1 inhibition, pregnane X receptor (PXR)/RXR activation, liver X receptor (LXR)/RXR activation and 1α, and 25-dihydroxyvitamin D3 (vitamin D3) receptor (VDR)/RXR activation were specifically associated with the non-disease-modulated NRs. These results have provided novel insight on the potential functions of various NRs in modulating the pathogenesis of human autoimmune arthritis and metabolic diseases.

Since we found that upregulation and downregulation of certain NRs can be shared in several human diseases (Fig. 6a), we examined whether the NRs shared in human diseases can be used as biomarkers for the diseases and complications. To test this issue, we organized the analysis results in Fig. 6e. The results showed that upregulation of three NRs such as NR1H3, NR1I1, and NR3A1 can be used as biomarkers for rheumatoid arthritis and that upregulation of NR1I1 alone and downregulation of only two NRs NR1A1 and NR3C3 can be used as potential biomarkers for rheumatoid arthritis with familial hypercholesterolemia. In addition, upregulation of NR3C4 can be used as potential biomarker for type 2 diabetes whereas downregulation of two NRs including NR3C3 and NR4A3 can only be used as biomarkers for diabetes with rheumatoid arthritis as a complication. Moreover, upregulation of three NRs such as NR4A1, NR4A2, and NR4A3 can be used for the potential biomarkers for obesity while downregulation of NR1D1 can be used as a potential biomarker for obesity with rheumatoid arthritis as a complication. Finally, downregulation of NR1A1 alone can be used as the biomarker for obesity complicated with rheumatoid arthritis and familial hypercholesterolemia. The results suggest that the NRs shared in human diseases may be highly valuable in serving as potential biomarkers for detection of autoimmune arthritis, metabolic diseases, and their complications.

### The expression of nuclear receptors are regulated by numerous inflammation-modulating pathways and mitochondrial energy metabolic enzymes

We then hypothesized that the expression of NRs is regulated by numerous inflammation-modulating pathways. To test this hypothesis, we examined the expression of NRs in various gene-deficient mouse models and cells with overexpression of genes of interests. *First*, two NRs such as Nr1h3 and Nr1i1 were found to be upregulated, and four other NRs were downregulated in the aortic arch in apolipoprotein E (ApoE)−/− mice fed with 24 weeks of high fat diet (Table 15). However, only one NR, Nr2f1, was found to be downregulated in the aortic arch of ApoE−/− mice fed with 8 weeks of high fat diet. These findings suggest that NR modulation in ApoE−/− in the aortic arch requires prolonged high fat diet feeding.

**Table 15:** A large nuclear receptor is downregulated in proatherogenic mouse models ApoE−/−, LDL-R−/−, and type 2 diabetes mouse model db/db

Disease	Tissue/cell type	Number		Upregulated gene	Fold*	Downregulated gene	Fold*	PMID/GEO ID
		Up	Down		∆		∆	
ApoE−/− 8-week HFD	Aortic arch	0	1			Nr2f1	−1.2	20577049/GSE18443
ApoE−/− 24-week HFD		2	4	Nr1h3	1.26	Nr1a2	−1.25	
				Nr1i1	1.27	Nr1b2	−1.2	
						Nr1d1	−1.28	
						Nr3a1	−1.22	
LDL-R−/− VS. WT	Macrophages of aorta	2	9	Nr1c3	1.29	Nr1a2	−1.3	21868699/GSE24342
				Nr3a1	1.3	Nr1b2	−1.2	
						Nr1d2	−1.28	
						Nr1i1	−1.3	
						Nr2b3	−1.25	
						Nr3c1	−1.2	
						Nr3c4	−1.27	
						Nr4a1	−1.32	
						Nr4a2	−1.28	
db/db VS. WT	Glomerular endothelial cell	1	3	Nr1b2	1.74	Nr1i1	−1.47	20706631/GSE21324
						Pgr	−1.52	
						Ar	−1.67	

Abbreviations: *WT* wild type, *ApoE−/−* apolipoprotein E-deficient mice; *LDL-R−/−* low-density lipoprotein receptor deficient mice, *db/db mice* leptin receptor gene mutant mice, *HFD* high fat diet, *VS.* versus

*Fold change > 1.2

In another study, it was shown that expression of NRs were upregulated and nine NRs were downregulated in aortic macrophages of low-density lipoprotein receptor (LDL-R)-deficient mouse aortic macrophages relative to wild type (Table 15). In a separate study, one NR Nr1b2 was found to be upregulated but three NRs were downregulated in diabetic *db/db* glomerular endothelial cells (Table 15). These results suggest that once again in proatherogenic models and type 2 diabetes model, there is a less tendency for NRs to be upregulated than downregulated.

*Second*, we examined whether inflammatory cytokine signaling pathways can downregulate NR expression. In Table 16, in interferon-γ (IFN-γ)-stimulated endothelial cells, interleukin-1β (IL-1β)-stimulated endothelial cells, IL-1β-stimulated human peripheral mononuclear cells (PBMCs) and tumor necrosis factor-α (TNF-α)-stimulated PBMCs, the NR expressions were either upregulated and downregulated in similar numbers or less upregulated than downregulated.

**Table 16:** Pro-inflammatory cytokine signaling negatively regulates the expression of nuclear receptors

Disease		Tissue/cell type	Number		Upregulated gene	Fold*	Downregulated gene	Fold*	PMID/GEO ID
			Up	Down		∆		∆	
IFN-γ stimulation		EC	2	2	NR2F1	1.39	NR2B1	−1.26	19553003/GSE3920
					NR3C1	1.53	NR2F6	−1.36	
IL-1β stimulation		EC	3	7	NR1H4	5.32	NR1B2	−3.84	21469100/GSE19240
					NR3A1	2.21	NR1B3	−4.93	
					NR5A2	6.57	NR1F1	−4.72	
							NR1F2	−2.58	
							NR3A2	−4.94	
							NR3B3	−11.03	
							NR4A1	−3.43	
Human PBMCs-IL-1β and TNF-α stimulations	IL-1β 2 h	PBMC	0	3			NR1A1	−14.93	23104095/GSE40838
							NR1I1	−5.1	
							NR3A2	−6.54	
	IL-1β 6 h		0	1			NR5A2	−23.26	
	TNF-α 2 h		1	1	NR1B2	38.32	NR2A1	−21.11	
	TNF-α 6 h		2	3	NR1B2	27.28	NR1A2	−23.92	
					NR3B3	4.92	NR1B3	−16.22	
							NR4A1	−3.86	

Abbreviations: *IFN-γ* interferon gamma, *IL-1β* interleukin-1β, *TNF-α* tumor necrosis factor-α-like, *PBMCs* peripheral blood mononuclear cells

*Third*, in Table 17, we examined whether anti-inflammatory cytokine pathways and inhibition of the pro-inflammatory transcription factor regulate NR expressions. We observed that NR expressions were modulated in hepatocellular carcinoma cells stimulated with transforming growth factor-β (TGF-β), palatal mesenchyme cells from TGF-β knockout (KO) mice, in conventional T cells stimulated with anti-CTLA-4 (cytotoxic T-lymphocyte-associated protein 4, also known as CD152, a T cell co-suppressor) antibody, in regulatory T cells (Tregs) stimulated with anti-CTLA-4 antibody and in hearts extracted from cardiac-specific transgenic PPARα mice. In a study with NF-kB inhibitor-treated cells, six NRs were upregulated and five NRs were downregulated. These results demonstrated that immune suppressor pathways CTLA-4, NF-kB inhibitor, and Treg suppress inflammation by significantly upregulating the expression of nine NRs including NR4A1 (5.6–8.3 folds), NR4A2 (6.7–15.8 folds), NR4A3 (4.6–8.1 folds), NR1B2 (6.7 folds), NR1D1 (3.4 folds), NR2A2 (3.2 folds), NR1H4 (2.7 folds), NR2C1 (5.4 folds), and NR3A2 (4.2 folds).

**Table 17:** Anti-inflammatory cytokine signaling and Tregs positively regulate the expression of nuclear receptors

Disease		Tissue/cell type	Number		Upregulated gene	Fold*	Downregulated gene	Fold*	PMID/GEO ID
			Up	Down		∆		∆	
TGF-β stimulation		HCC Huh-7 cells	8	7	NR1B1	1.19	NR1B2	−1.21	19723656/GSE10393
					NR1H2	1.37	NR1C3	−1.23	
					NR2B1	1.75	NR1D2	−1.35	
					NR2B2	1.23	NR1F1	−1.42	
					NR2F1	1.27	NR1H4	−1.45	
					NR2F2	1.36	NR3C1	−1.21	
					NR2F6	1.37	NR5A2	−1.29	
					NR0B2	1.59			
TGF-β KO		PM cells	2	3	NR2C2	1.21	NR2C1	−1.26	23975680/GSE46150
					NR3C1	1.23	NR2F6	−1.3	
							NR3C4	−1.67	
Tconv stimulate by anti-CTLA-4		Spleen and lymph node	7	3	NR1D2	1.69	NR1B3	−1.14	23277554/GSE42267
					NR1H3	1.2	NR2B1	−1.18	
					NR2C2	1.23	NR2C1	−1.28	
					NR3C4	1.64			
					NR4A1	5.61			
					NR4A2	15.78			
					NR4A3	8.12			
Treg stimulated with anti-CTLA-4		Spleen and lymph node	5	4	NR1D2	1.53	NR1B1	−1.25	23277554/GSE42267
					NR1F1	2.4	NR1B3	−1.32	
					NR3C4	2.36	NR1F1	−1.23	
					NR4A1	3.3	NR3A1	−1.23	
					NR4A2	6.67			
Cardiac-specific transgenic (Tg-PPARα) mice		Heart	5	13	NR1F2	1.22	NR1A1	−1.27	22055503/GSE33101
					NR1H3	1.46	NR1C1	−1.67	
					NR1I2	0.83	NR1F1	−3.84	
					NR2A1	1.24	NR1F3	−2	
					NR0B2		NR2B1	−1.22	
							NR2B2	−1.22	
							NR2B3	−1.59	
							NR2F2	−1.22	
							NR2F6	−1.27	
							NR3B1	−1.22	
							NR3B2	−1.23	
							NR3C1	−1.25	
							NR3C3	−1.27	
NFκB inhibitor	4 h	EKC	6	5	NR1B1	2.33	NR1C2	−2.81	15722350/GSE2489
					NR1B2	6.73	NR1F1	−6.54	
					NR1D1	3.36	NR1I3	−2.93	
					NR2A2	3.16	NR3A2	−2.91	
					NR2C1	2.01	NR5A2	−5.17	
					NR4A1	8.34			
	48 h		4	4	NR1H4	2.71	NR1C3	−5.46	
					NR2C1	5.35	NR2A1	−3.63	
					NR3A2	4.2	NR2F1	−14.03	
					NR4A3	4.56	NR3B3	−4.82	

Abbreviations: *HCC* hepatocellular carcinoma cells, *TGF-β* transforming growth factor-β, *PM* palatal mesenchyme, *Tconv* conventional T cells, *Treg* regulatory T cell, *EKC* epidermal keratinocytes

*Fourth*, in Table 18, we examined whether a key enzyme of tricarboxylic acid (TCA) cycle, isocitrate dehydrogenase (IDH), regulates the expression of nuclear receptors. The results showed that IDH mutation in isogenic epithelial cells results in significant upregulation of six NRs and downregulation of eight NRs. *Fifth*, in Table 19, we examined whether four key enzymes of the mitochondrial respiratory chain including nicotinamide adenine dinucleotide (quinone) (NADH) dehydrogenase (Nd2), succinate dehydrogenase (SDH), cytochrome c oxidase-4 (COX4), and mitochondrial respiratory chain complex IV regulate the expression of nuclear receptors. The results showed that the mutations of these enzymes result in significant changes in the expression of NR2F2 (Nd2 mutation induced sevenfold upregulation), NR5A2 (Nd2 mutation induced 27-fold downregulation), NR1A2 (COX4 mutation induced 46.5-fold upregulation), and NR2F6 (Cox4 mutation induced 3.3-fold downregulation). Taken together, these results suggest that the expressions of nuclear receptors are regulated by numerous inflammation-modulating pathways and mitochondrial energy metabolic enzymes.

**Table 18:**
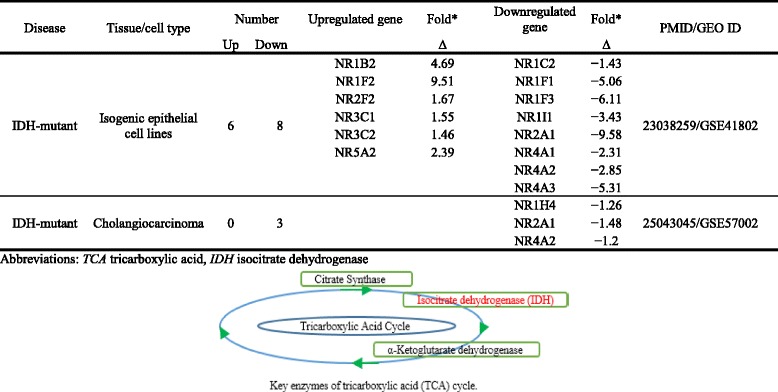
A key enzyme of tricarboxylic acid (TCA) cycle, isocitrate dehydrogenase, regulates the expression of nuclear receptors

**Table 19:**
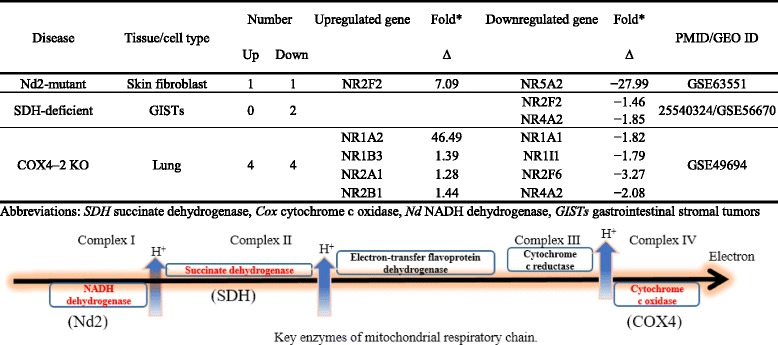
Key enzymes of the mitochondrial respiratory chain regulate the expression of nuclear receptors

### Innate immune sensor inflammasome/caspase-1 pathway plays a critical role in regulating the expression of most nuclear receptors

In Fig. 4, we found that innate immune sensor PRRs such as NOD1, NOD2, NOD4, and IFI16 may either act as upstream regulators or downstream targets of NRs in tissues and that the NLR-mediated regulation on NR expression in tissues are evolutionally conserved and mainly act toward suppression of NRs during microbial infection-triggered inflammations. To consolidate this finding, we determined whether gene deficiencies of caspase-1 and other inflammasome components affect the expression of NRs.

As shown in Table 20, deficiency of caspase-1 in ApoE−/− mouse aorta, adipose tissue, deficiency of caspase-1 in associated speck-like protein containing a CARD (ASC)−/− background, deficiency of histone deacetylase and caspase-1 substrate sirtuin 1 (Sirt1) in Tregs, and deficiency of NLRP3 in adult and children PBMCs led to mostly upregulation of NRs instead of downregulation of NRs. For example, the deficiency of NLRP3 led to upregulation of 26 NRs (54%) but downregulation of 9–11 NRs (19–23%). These results suggest that caspase-1/NLRP3 inflammasome pathways play a critical role in regulating the expression of NRs. In addition, in Table 21, we also noticed that the inflammasome/caspase-1 deficiencies upregulated 29 NRs (60%), downregulated 10 NRs (21%), but did not change the expression of 9 NRs (19%).

**Table 20:** Nuclear receptors are significantly changed in caspase-1 and Sirt1 knockout mice, indicating that caspase-1-Sirt1 pathway negatively regulates nuclear receptor expression

Disease		Tissue/cell type	Number		Upregulated gene	Fold*	Downregulated gene	Fold*	PMID/GEO ID
			Up	Down		∆		∆	
AopE−/−/Casp1−/− vs. ApoE−/−		Aorta	3	0	NR1A2	1.22			GSE72448
					NR1D2	1.17			
					NR2C2	1.19			
		Adipose	10	1	NR1A1	1.35	NR6A1	−1.32	
					NR1C3	1.84			
					NR1F1	1.78			
					NR1I3	1.22			
					NR2A1	2.24			
					NR2B1	1.52			
					NR3A1	1.39			
					NR3C1	1.41			
					NR3C4	1.66			
					NR4A2	1.16			
Casp1−/−/ASC−/− vs. ASC−/−		White adipose tissue	7	0	NR1A1	1.35			21876127/GSE25205
					NR1C3	1.84			
					NR1I3	1.22			
					NR2A1	1.17			
					NR2B1	1.52			
					NR3C4	1.65			
					NR4A2	1.16			
Sirt1−/− vs. WT		Treg	4	0	NR1B1	1.2			21199917/GSE26425
					NR1F1	1.29			
					NR2F6	1.22			
					NR4A3	1.29			
Cardiac-specific transgenic (Tg-Sirt1) mice		Heart	2	6	NR3C4	1.52	NR1B1	−3.57	22055503/GSE33101
					NR0B2	1.35	NR1B3	−1.25	
							NR1F3	−2	
							NR1I1	−1.27	
							NR2B3	−1.22	
							NR2C1	−1.22	
NLRP3 mutation	Adult control	PBMC	26	9	NR1B1	1.22	NR1F1	−2.93	–/GSE43553
					NR1B2	1.47	NR1F2	−1.4	
					NR1B3	1.43	NR2C1	−1.93	
					NR1C2	1.18	NR3C1	−1.38	
					NR1C3	1.2	NR3C2	−1.68	
					NR1I1	1.73	NR4A1	−1.32	
					NR1I2	1.2			
					NR2A1	1.53			
					NR2B3	1.25			
					NR2E1	1.31			
					NR2E3	1.57			
					NR2F1	1.2			
					NR3A1	1.61			
					NR3A2	1.28			
					NR3B1	1.34			
					NR4A3	1.27			
					NR5A1	1.42			
					NR6A1	1.3			
					NR0B2	1.34			
	Children control		26	11	NR1A1	1.4	NR1D1	−1.82	
					NR1A2	1.24	NR1D2	−1.56	
					NR1B1	1.31	NR1F1	−2.53	
					NR1B2	1.51	NR2B2	−1.44	
					NR1B3	1.49	NR2C1	−2.25	
					NR1C3	1.32	NR2C2	−1.29	
					NR1I1	1.69	NR3C1	−1.3	
					NR1I2	1.27	NR3C2	−1.53	
					NR2A1	1.66	NR4A1	−1.37	
					NR2A2	1.22	NR4A2	−4.23	
					NR2E1	1.21			
					NR2E3	1.55			
					NR2F1	1.24			
					NR2F6	1.26			
					NR3A1	1.23			
					NR3A2	1.41			
					NR3B1	1.65			
					NR3C3	1.21			
					NR4A3	1.23			
					NR5A1	1.41			
					NR6A1	1.29			
					NR0B2	1.34			

Abbreviations: *ApoE−/−* apolipoprotein E-deficient mice, *Casp1−/−* caspase-1-deficient mice, *HFD* high fat diet, *ASC−/−* PYD and CARD domain-containing deficient mice, *Sirt 1−/−* sirtuin 1-deficient mice; *WT* wild-type mice, *NLRP3* NLR family pyrin domain containing 3 deficient mice; *vs.* versus

**Table 21:**
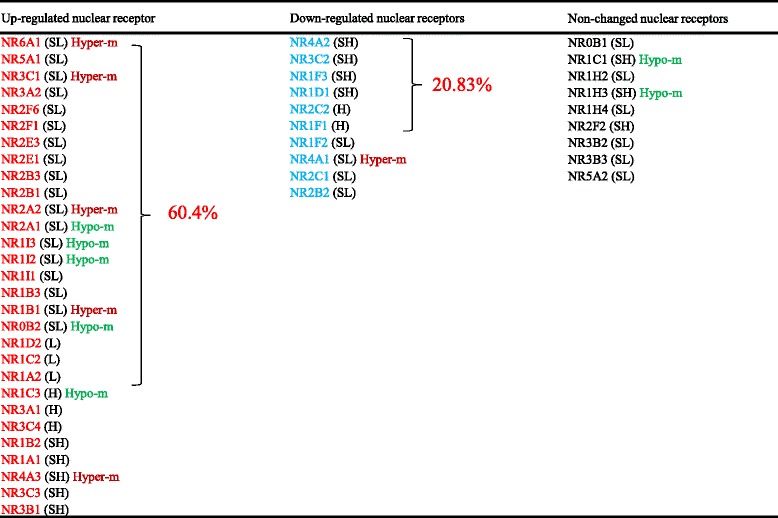
The expression changes of NRs in the presence of inflammasome/caspase-1 deficiencies

Moreover, we noticed that among top 10 pathways identified with the Ingenuity Pathway Analysis for the inflammasome/caspase-1 deficiency-upregulated NRs, three pathways including aryl hydrocarbon receptor signaling, RAR activation, and estrogen receptor signaling were unique. Similarly, among the top 10 pathways identified with the Ingenuity Pathway Analysis for the inflammasome/caspase-1 deficiency-downregulated NRs, seven pathways including circadian rhythm signaling, thyroid cancer signaling, Nur77 signaling in T lymphocytes, calcium-induced T-lymphocyte apoptosis, melatonin signaling, T helper cell differentiation, and non-small cell lung cancer signaling were specific. Furthermore, among the top 10 pathways identified with the Ingenuity Pathway Analysis for the inflammasome/caspase-1 deficiency-non-changed NRs, three pathways including LPS/IL-1-mediated inhibition of RXR function, LXR/RXR activation, and Toll-like receptor signaling were unique. Therefore, these pathways may not play a significant role in progression of inflammatory pathologies. Taken together, these results suggest that inflammasome/caspase-1 pathway deficiencies regulate the expressions of most NRs (81%) and that inflammasome/caspase-1 innate immune sensors control the expression of most NRs.

### We propose a new paradigm that most nuclear receptors are anti-inflammatory HAMPs for regulating the balance of inflammation, inhibition of inflammation, and resolution of inflammation

HAMPs (homeostasis-associated molecular pattern molecules), the new concept we proposed, are designated for mitigating the progression of inflammation or inhibition of inflammation under sterile inflammation. These HAMP receptors initiate anti-inflammatory/homeostatic signaling and promote inflammation resolution [5]. Since most endogenously metabolite-nuclear receptor signals inhibit inflammation and maintain the tissue homeostasis, we propose that most NRs act as HAMP receptors. To consolidate this new hypothesis, we conducted an extensive literature search (Parts 2, 3, and 4 in Fig. 1). We found the following supporting evidences.

*The* ***first*** *supporting evidence* for classifying most of nuclear receptors as HAMP receptors is presented in Tables 8, 9, and 10 (Part 2 in Fig. 1): (1) Mutations in NR significantly increase the risk for development of human metabolic diseases (Tables 4, 5, 6, and 7), suggesting that NR sequence changes may weaken the NR functions in suppressing human metabolic diseases and inflammation; (2) NR deficiencies lead to abnormal mouse phenotypes and inflammation from the MGI database (Tables 8 and 9), suggesting that NRs’ expression and functions are essential for maintaining the homeostasis and inhibition of inflammation and that NR deficiencies increase the likelihood of developing metabolic diseases in mice and potentially in humans.

*The* ***second*** *supporting evidence* for classifying most of nuclear receptors as HAMP receptors is presented in Table 12 (Part 3 in Fig. 1). NRs inhibit inflammation signaling gene functions and inflammation readouts. Of note, 12 out of 15 NRs have anti-inflammatory roles verified by published papers (Table 12).

*The* ***third*** *supporting evidence* for classifying most NRs as HAMP receptors is demonstrated in Tables 13, 14, and 15 (Part 4 (1) and (2) in Fig. 1). NRs were less upregulated than downregulated during the progression of metabolic, cardiovascular, and autoimmune diseases and cancers, suggesting that NRs’ physiological expression and functions may block the pathogenesis and progression of those diseases.

*The* ***fourth*** *supporting evidence* for classifying most of nuclear receptors as HAMPs is demonstrated in Tables 16 and 17 (Part 4 (3) in Fig. 1). Inflammation signaling genes regulated nuclear receptor expression levels as judged by the following results: (1) Most NRs were downregulated when stimulated with pro-inflammatory agents, suggesting that the pro-inflammatory signals suppress the NRs expression, and (2) some NRs were downregulated when anti-inflammatory signaling genes were deficient. In contrast, those NRs were upregulated when anti-inflammatory signals were activated.

*The* ***fifth*** *supporting evidence* for classifying most of nuclear receptors as HAMP receptors is demonstrated in Tables 20, 21, and 22 (Part 4 (4) in Fig. 1). Most NRs were more upregulated than downregulated when innate immune sensor inflammasome/caspase-1 genes were deficient. In contrast, caspase-1-degrading gene histone deacetylase Sirt1 [8] transgene may have anti-inflammatory functions by increasing the expression of certain NRs.

**Table 22:**
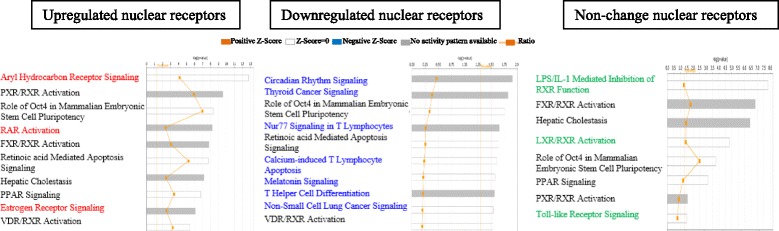
Signal pathways that are upregulated by genes listed in Table 21

## Discussion

NRs are a class of 48 lipophilic ligand-activated transcription factors identified as key players of metabolic and developmental processes. Upon activation by the ligand messenger, NRs typically function as transcription factors where they bind to recognition elements on the genomic DNA and regulate the expression of target genes via type I, II, and III signaling formats [66]. Regardless of the significant progress that has been made in characterizing NR functions and expression, the global profiling of NR expression in human immune and cardiovascular tissues and potential mechanisms underlying the physiological expression of NRs remained poorly defined. In addition, the important issue of how innate immune sensor inflammasome/caspase-1 and other inflammatory signaling globally regulate NR expression in tissues and cells also remained unknown. To examine these issues, we took panoramic profiling database analysis approaches and made the following important findings: (1) NRs are differentially expressed in human and mouse tissues and NR expression may be under regulation by oxygen sensors, angiogenesis pathway, stem cell master genes, PRRs, and tissue hypomethylation/hypermethylation indices; (2) NR sequence changes and mutations are associated with increased risks for development of metabolic diseases, cardiovascular diseases, hormone insensitivity/resistance, cancers, and autoimmune diseases; (3) NRs have less tendency to be upregulated than downregulated in human autoimmune diseases, metabolic diseases, and cancers, which may be regulated by numerous inflammation-modulating pathways and mitochondrial energy metabolic enzymes; (4) The innate immune sensor inflammasome/caspase-1 pathway plays a critical role in regulating the expression of most NRs (Table 23); and (5) We propose a new paradigm that most NRs are anti-inflammatory HAMPs for regulating the balance of inflammation, inhibition of inflammation, and resolution of inflammation.

**Table 23:** Nuclear receptor expression was regulated by ApoE and LDL-R, pro/anti-inflammatory cytokines, and inflammasomes in pathology

Human metabolic disease	NRNC symbol	ApoE KO	LDL-R KO	IFN-γ stimulation	IL-1β stimulation	TNF-α stimulation	NFκB inhibitor	TGF-β KO	Tg-PPARα	CAS1 KO	ASC KO	Tg-Sirt1	NLRP3 mutant
Upregulation (from Fig 6c)	NR0B1												
	NR1B1						↑					↓	↑
	NR1B3				↓	↓						↓	↑
	NR1F3								↓			↓	
	NR1I1	↑	↓									↓	↑
	NR2F6			↓				↓	↓				↑
	NR3B2								↓				
	NR4A1		↓		↓	↓	↑						↓
	NR5A1												↑
	NR4A2		↓								↑		↓
	NR6A1									↓			↑
	NR4A3						↑						↑
Downregulation (from Fig 6c)	NR1A1								↓	↑	↑		↑
	NR1B2	↓	↓		↓	↑	↑						↑
	NR1C1								↓				
	NR1D1						↑						↑
	NR1H3	↑							↑				
	NR3C3								↓				↑
	NR1F1				↓		↓		↓	↑			↓
	NR1C3		↑				↓			↑	↑		↑
	NR3A1	↓	↑		↑					↑			↑
	NR2C2							↑		↑			↓
	NR3C4		↓					↓		↑	↑	↑	
	NR1F2				↓				↑				↓
	NR3B3				↓	↑	↓						
	NR3C1		↓	↑				↑	↓	↑			↓
	NR2A2						↑						↑
	NR2B3		↑						↓			↓	↑
	NR2C1						↑	↑				↓	↓
	NR2E3												↑
	NR2F1	↓		↑			↓						↑
	NR3A2				↓		↑						↑

Abbreviations: *KO* knockout, *ApoE* apolipoprotein E, *LDL-R−/−* low-density lipoprotein receptor, *IFN-γ* interferon gamma, *IL-1β* interleukin 1 beta, *TNF-α* tumor necrosis factor-α-like; *TGF-β* transforming growth factor-β, *Casp1−/−* caspase-1-deficient mice; *ASC−/−* PYD and CARD domain-containing deficient mice, *Tg-Sirt1* transgenic sirtuin 1 mice, *NLRP3* NLR family pyrin domain containing 3 deficient mice, *PBMC* peripheral blood mononuclear cells

We utilized an experimental database mining approach that was pioneered and developed in our laboratory throughout the years [2, 67–69]. By analyzing DNA sequencing data from tissue cDNA libraries, we were able to study expression profiles of NRs in various tissues. Since the gene expression sequencing tag (EST) data deposited in the NIH-NCBI-UniGene database have been established based on DNA sequencing data, the data extracted from EST database mining are more precise in providing the tissue expression profiles of genes than traditional hybridization- and primer annealing-based approaches like Northern blots and RT-PCRs [2]. Of note, since the UniGene database does not have many non-tumor cell line-related gene expression data in the presence of various gene deficiencies and stimulation conditions, we analyzed microarray-based gene expression data deposited in NIH-GEO datasets to determine NR expression changes under pathological conditions. Also, as all the data we provided in this manuscript were collected from cDNA cloning, DNA sequencing experiments, and microarray datasets rather than theoretical data derived from computer modeling, we believe that our findings are relevant for many biological and pathological scenarios. Nevertheless, herein we acknowledge that further well-designed experiments are needed to consolidate our findings.

As we pointed out in Table 7, a previous paper reported a mouse NR tissue expression profile using nucleic acid binding based RT-PCR technique [26, 27]. However, NR superfamily expressions using a more accurate DNA sequencing-based technique have not been profiled for human tissues. Other reports have confirmed the tissue distribution of few NRs. For example, as previously mentioned, the rat tissue distribution and/or the relative level of NR3A1 and NR3A2 expression seems to be quite different, i.e., moderate to high expression in the uterus, testis, pituitary, ovary, kidney, epididymis, and adrenal for NR3A1 and the prostate, ovary, lung, bladder, brain, uterus, and testis for NR3A2 [70]. Another study showed that NR2E3 mRNA was detected in the adrenal gland, thyroid gland, prostate, testis, uterus, trachea, and salivary gland [71]. A study assessed the expression patterns of NRs in peripheral blood mononuclear cells and found that 33/48 NRs were expressed in peripheral blood mononuclear cells [72]. In order to clearly summarize our findings, study the expression profile of NRs, and offer a simple, powerful way to obtain highly relational information about their physiologic functions as individual proteins and as a superfamily, we proposed a novel pyramid model to highlight several categories of NR activities in many important tissues. This pyramid model is significant as it improves our understanding of the tissue differences of NR machinery. This model is also significant for understanding the potential pharmacological side effects of new drugs targeting NRs in those tissues. Based on the different distributions and relative levels of the NRs in different target tissues, ligands could be used to elicit beneficial hormone-like activities and reduce adverse side effects of NR-targeted drugs.

The current DAMP receptor model emphasizes only the danger signals generated from endogenous metabolic processes. It fails to recognize the roles of potential endogenous metabolites in anti-inflammatory responses, inflammation resolution, and maintenance of homeostasis. As we pointed out in our previous report [5], it is significant for us to address these limitations and shift the paradigm to form a new model [73] to recognize novel anti-inflammatory and homeostatic signals derived from endogenous metabolites. Recent advances in immunology have clearly demonstrated the well-published “two arms model.” This model states that in addition to the pro-inflammatory immunoeffector and T cell co-stimulatory mechanisms, there are several immunotolerance and anti-inflammatory mechanisms mediated by the immune system. These anti-inflammatory mechanisms include T cell co-inhibition/co-suppression pathways, T cell anergy, regulatory T cells [74], and secretion of anti-inflammatory/immunosuppressive cytokines such as transforming growth factor-β (TGF-β), interleukin-10 (IL-10), IL-35 [69, 75], and IL-37 as we and others reported, etc. We have reported two types of lysophospholipids such as lysophosphatidylserine (LysoPS) and lysophosphatidylethanolamine (LPE) [5] and a few uremic toxins as anti-inflammatory homeostasis-associated molecular patterns [76]. In addition, along the same line, endogenous specialized pro-resolving mediators have been identified as regulators of infection and inflammation [77].

Our new classification of most NRs as homeostasis-associated molecular pattern (HAMP) receptors was that some NRs have been experimentally proved to bind promiscuously to certain types of “patterns” but not exclusively stick to highly specific ligands (Table 3). For example, activation of NRs by a variety of endo- and exogenous chemicals are elemental to induction and repression of drug-metabolism pathways. The master xenobiotic-sensing NRs, the promiscuous pregnane X receptor (PXR), and less-promiscuous constitutive androstane receptor (CAR) are crucial to initial ligand recognition, jump-starting the metabolic process [78]. In addition, phytoestrogens are natural endocrine disruptors that interfere with estrogenic pathways. They insert directly within the hormone-binding domain of estrogen receptor-α (ER-α) and β, with a preference for the β isoform of which the concentration predominates in the normal mammary epithelium [79]. Moreover, bisphenol A (BPA) is widely used as a component in polycarbonate plastics for food and beverage packaging, epoxy linings for canned foods, and dental sealants, among other applications. Experimental literature demonstrates BPA’s affinity for estrogen receptors and downstream effects on estrogen-responsive genes [80]. Those examples have clearly demonstrated that some NRs can promiscuously bind to certain types of “patterns” but not exclusively stick to highly specific ligands.

However, little is known how and why some receptors such as PXR and CAR develop promiscuity. The most widely accepted speculation is that both narrow and broad specificity seen for receptors or proteins are a result of natural selection process [81]. Less specificity of receptors provides evolutionary advantage to organisms that had to conduct a broad set of biological activities with limited protein repertoire and also allowed the organisms to evolve new responses to many endogenous and external ligands [82–84]. Promiscuity of such receptors complicate identification of the physiological ligands that activate them in vivo [85]. One way to identify candidate ligands for orphan NRs is to identify their three dimensional structure [86, 87]. However, receptor affinity for the ligand and the physiological concentrations of the ligand in the tissues have to be taken into account when determining the potential relevance of the specific ligand for the receptor function [85, 88]. Additionally, if it is known that the promiscuous NRs require intracellular lipid binding proteins to shuttle the ligand toward it (like PPAR utilizing certain FABPs—fatty acid binding proteins), the nuclear translocation of the particular protein in response to a compound can be used to determine potential ligands for the NR [85]. Nevertheless, identifying the potential endogenous ligands bound to the NR of interest in vivo by using mass spectroscopy, high-performance liquid chromatography (HPLC) or gas chromatography are the most relevant methods than the ones mentioned above [85, 89, 90].

We acknowledge that some of the fold changes shown in our tables are less than twofold. However, complex diseases such as diabetes, obesity, cancer, and autoimmune disorders are regulated by myriad of genes similar to quantitative traits [91, 92]. Previously, for most of the continuous traits, the strongest genetic association could explain only a small fraction of the genetic variance [93, 94]. However, later analyses revealed that casual loci with small effect size are also important in determining continuous traits and complex diseases such as schizophrenia [94]. Moreover, recent publications demonstrated that complex and chronic diseases are driven by accumulation of weak effects on the key genes and regulatory pathways [95, 96]. It is evident that polygenic effects are important across a wide variety of traits and diseases such as diabetes [97]. Therefore, it is our understanding that even a low fold change in potent transcription factors such as NRs can significantly impact progression of complex diseases.

## Conclusions

**Fig. 7:**
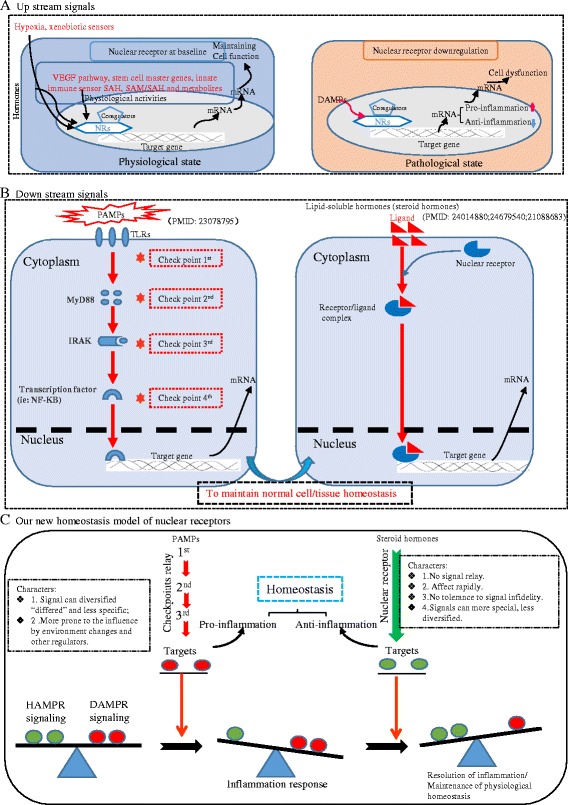
**a**–**c** Newly proposed working model which describes that most of the nuclear receptors can be classified as a family of homeostasis-associated molecular pattern receptors

To improve our understanding on most NRs as anti-inflammatory sensors and regulators, we propose a new working model and classified most NRs as homeostasis-associated molecular pattern receptors (HAMPRs) as shown in Fig. 7. *First*, NRs can sense lipophilic metabolites, hormones, and xenobiotics in a ligand-receptor-specific manner and “pattern” recognition manner; *second*, most NRs inhibit inflammation; *third*, a list of tissue homeostasis regulator pathways, tissue regeneration, and angiogenesis pathways including hypoxia sensors, VEGFR pathways, stem cell master regulators, and hypomethylation/hypermethylation may regulate the tissue expression of NRs (as shown in Fig. 7a); *fourth*, metabolic diseases [98, 99] and autoimmune arthritis have less tendency to upregulate than downregulate NR expression; *fifth*, comparing to conventional receptor-mediated pathways that signal via multiple steps for checking and relaying, NRs’ signals are much faster and require much less signal relay (as shown in Fig. 7b); and *sixth*, innate immune sensor inflammasome/caspase-1 pathways suppress the majority of NR expressions, suggesting that most NRs play critical roles in counteracting the role of DAMPs during sterile inflammatory pathologies and maintaining the homeostasis of tissues and cells in addition to their functions in metabolic, developmental [100], and growth processes [101–107]. Our new findings have significantly improved our understanding on NRs in the regulation of inflammation and tissue homeostasis (as shown in Fig. 7c).

## Additional files

Additional file 1: Figure S1. Nuclear receptors are differently expressed in human and mouse tissues. A: representative tissue mRNA distribution profile of housekeeping gene ARHGDIA in humans and Ldha in mice. See “Experimental Procedures” for details. B: mRNA distribution profiles of 26 nuclear receptors in 21 human tissues. C: MRNA distribution profiles of 15 nuclear receptors in 17 mouse tissues. The statistical significance was defined as when gene expression was larger than the upper limit of the confidence interval. Gene symbols are listed in Tables 1, 2, and 3. (PDF 296 kb)

Additional file 2: Figure S2. Forty-eight nuclear receptors are associated with SAH and SAM/SAH ratio in mouse tissues. A: shows the correlation of 48 nuclear receptors associated with SAH levels (hypomethylation status) in mouse tissues. B. shows the correlation of 48 nuclear receptors associated with SAM/SAH ratio in mouse tissues. (PDF 648 kb)

## Abbreviations

AIM-2: Absent in melanoma-2
ASC: Apoptosis speck-like CARD-containing protein
BPA: Bisphenol A
CARD8: Caspase recruitment domain family member 8
COX4: Cytochrome c oxidase-4
CTLA-4: Cytotoxic T-lymphocyte-associated protein 4
DAMP: Danger-associated molecular patterns
ER: Estrogen receptor
GWASs: Genome-wide association studies
HAMPs: Homeostasis-associated molecular patterns
HAMPRs: Homeostasis-associated molecular pattern receptors
HIF: Hypoxia-inducible factor
HMGB1: High-mobility group box 1
HPLC: High-performance liquid chromatography
IDH: Isocitrate dehydrogenase
IFI16: Interferon gamma-inducible protein 16
IL-1β: Interleukin-1β
KLF4: Kruppel-like factor 4
LPLs: Lysophospholipids
NOD: Nucleotide-binding oligomerization domain
NR: Nuclear receptor
Ox-LDL: Oxidized low-density lipoprotein
PAMPs: Pathogen-associated molecular patterns
PBMCs: Peripheral mononuclear cells
PHD2: Prolyl hydroxylase domain-containing protein 2
PRRs: Pattern recognition receptors
PXR: Pregnane X receptor
RAGE: Receptor for advanced glycation end products
RIG-1: Retinoid acid-inducible gene 1
SAH: S-adenosyl homocysteine
SAM: S-adenosyl methionine
SDH: Succinate dehydrogenase
TCA: Tricarboxylic acid
TCGA: The Cancer Genome Atlas
TGF-β: Transforming growth factor-β
TLR: Toll-like receptor
TNF-α: Tumor necrosis factor-α
Tregs: Regulatory T cells
VEGF: Vascular endothelial growth factor
