# Supplementary material for: A comprehensive data mining study shows that most nuclear receptors act as newly proposed homeostasis-associated molecular pattern receptors
Source: J Hematol Oncol. 2017 Oct 24;10:168. doi: 10.1186/s13045-017-0526-8 (PMC5655880; doi:10.1186/s13045-017-0526-8)
Supplement: Supplementary file 1 — Nuclear receptors are differently expressed in human and mouse tissues. A: representative tissue mRNA distribution profile of housekeeping gene ARHGDIA in humans and Ldha in mice. See “Experimental Procedures” for details. B: mRNA distribution profiles of 26 nuclear receptors in 21 human tissues. C: MRNA distribution profiles of 15 nuclear receptors in 17 mouse tissues. The statistical significance was defined as when gene expression was larger than the upper limit of the confidence interval. Gene symbols are listed in Tables 1, 2, and 3. (PDF 296 kb) [file 13045_2017_526_MOESM1_ESM.pdf]

Supplementary figure 1. Nuclear receptor are differently expressed in human and mouse tissues.
A. Representative tissue mRNA distribution profile of housekeeping gene ARHGDIA in humans and Ldha in mice

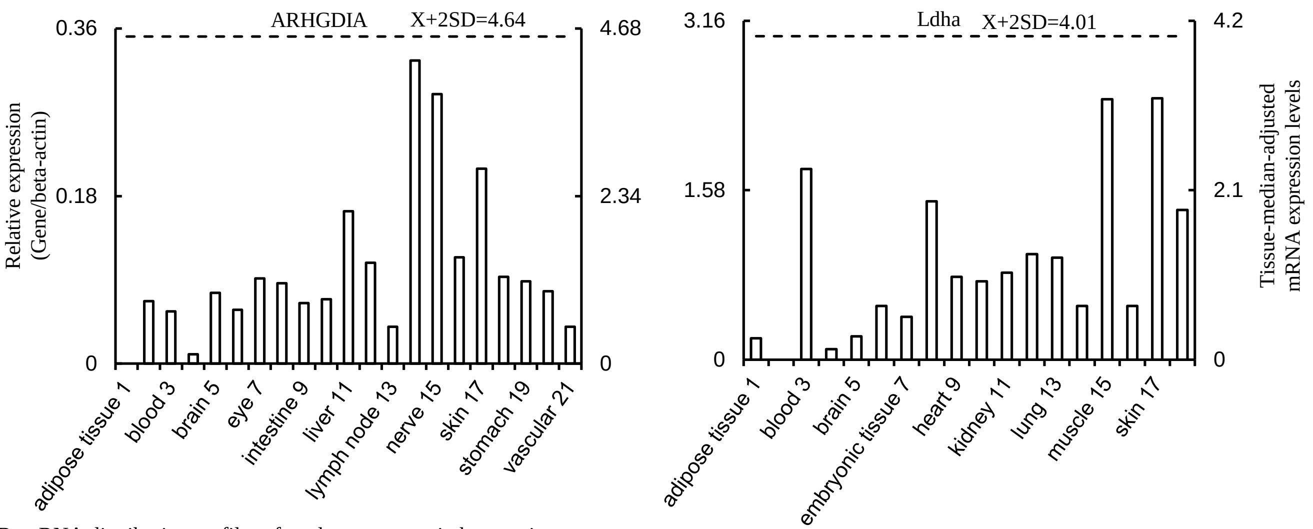

B. mRNA distribution profiles of nuclear receptors in human tissues

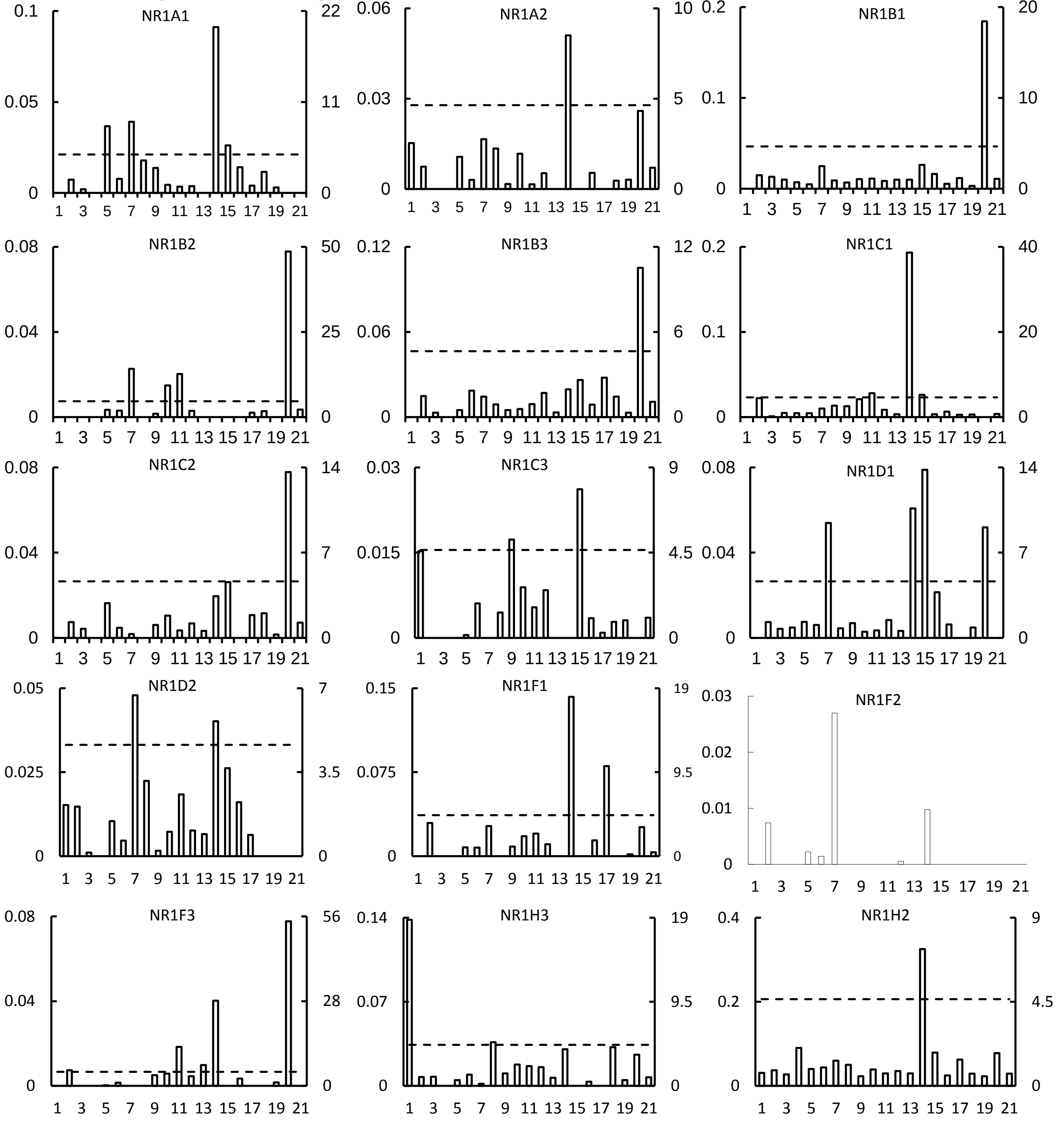

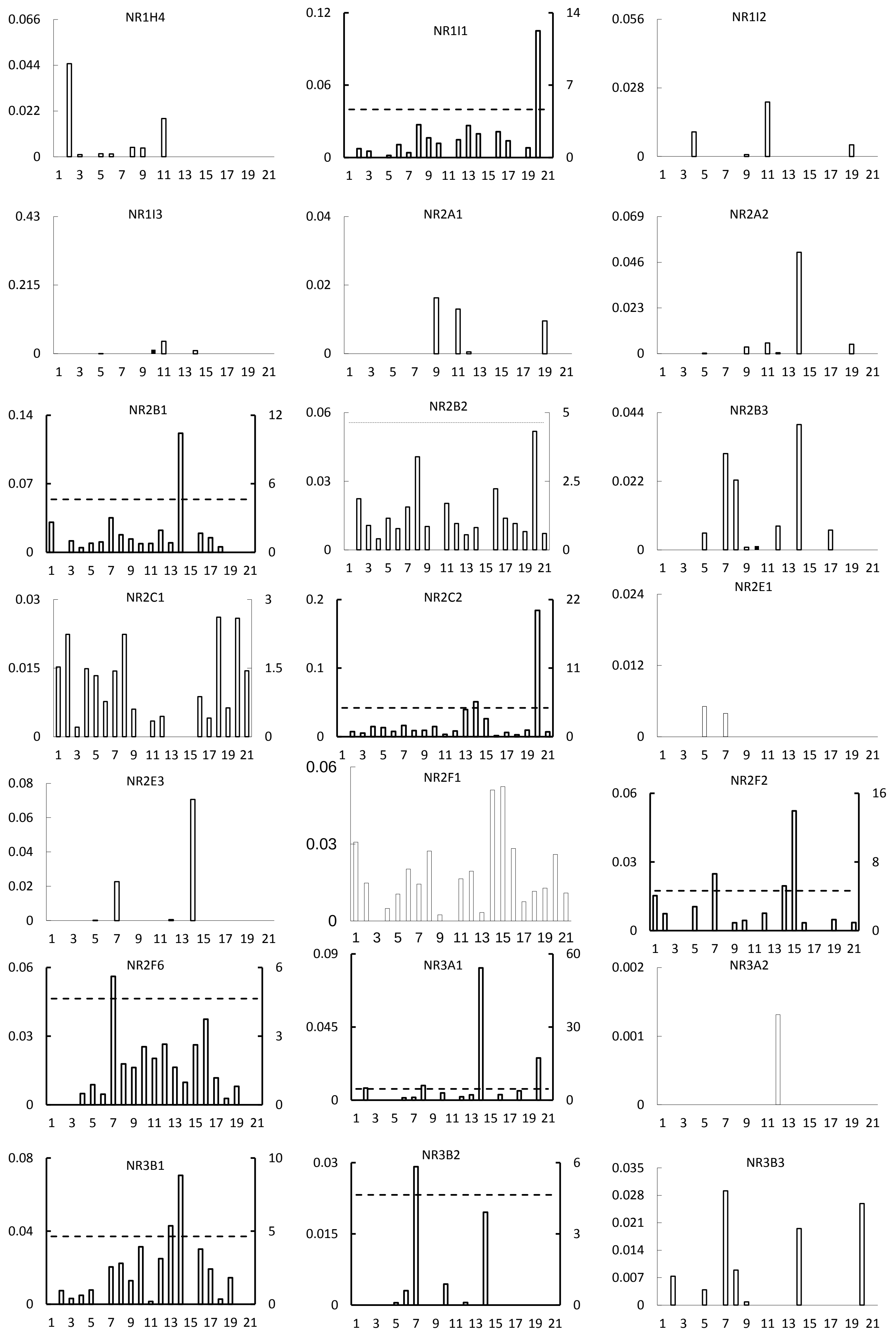
NR1H4
NR1I1
NR1I2
NR1I3
NR2A1
NR2A2
NR2B1
NR2B2
NR2B3
NR2C1
NR2C2
NR2E1
NR2E3
NR2F1
NR2F2
NR2F6
NR3A1
NR3A2
NR3B1
NR3B2
NR3B3

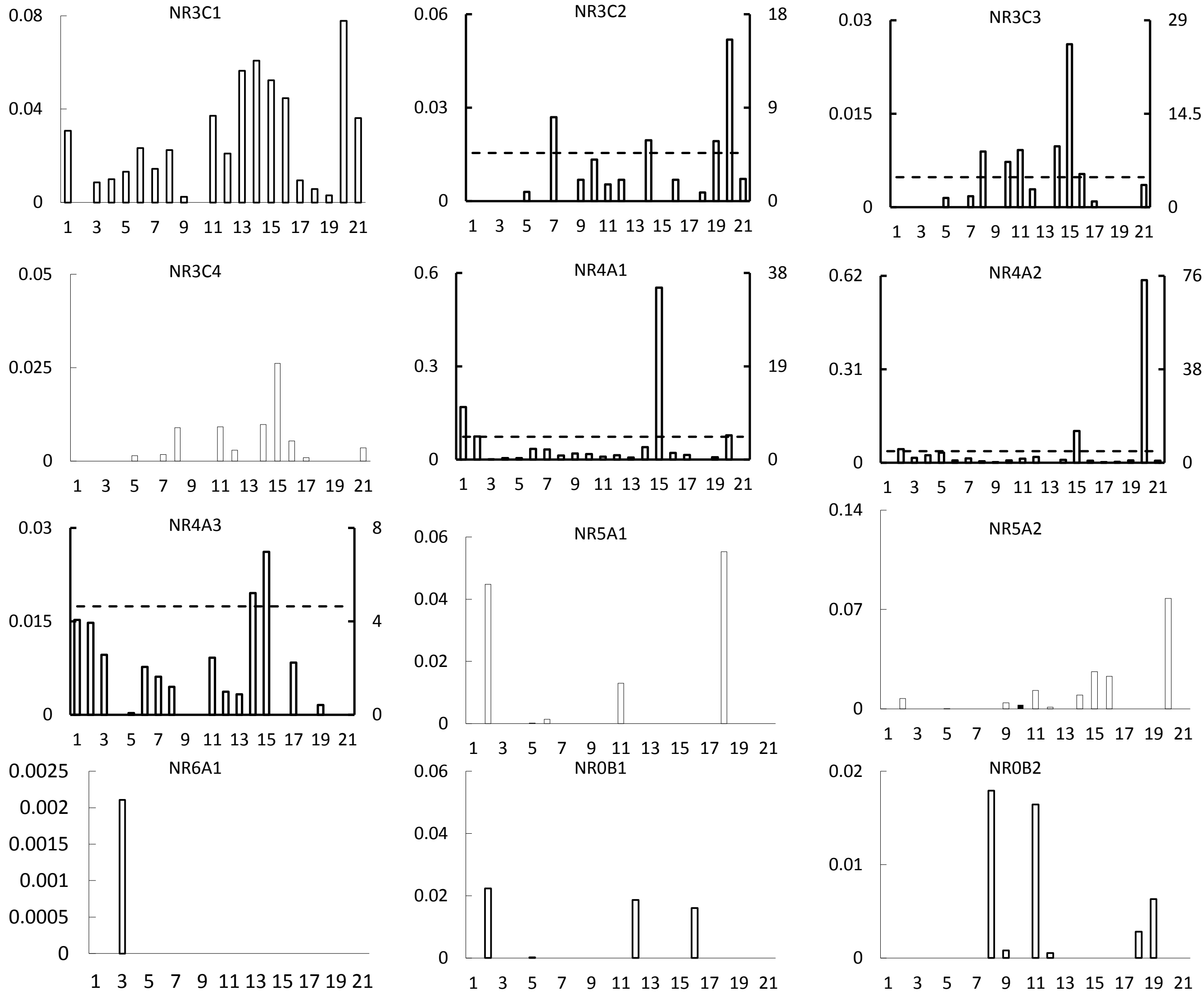

C. mRNA distribution profiles of nuclear receptors in mouse tissues

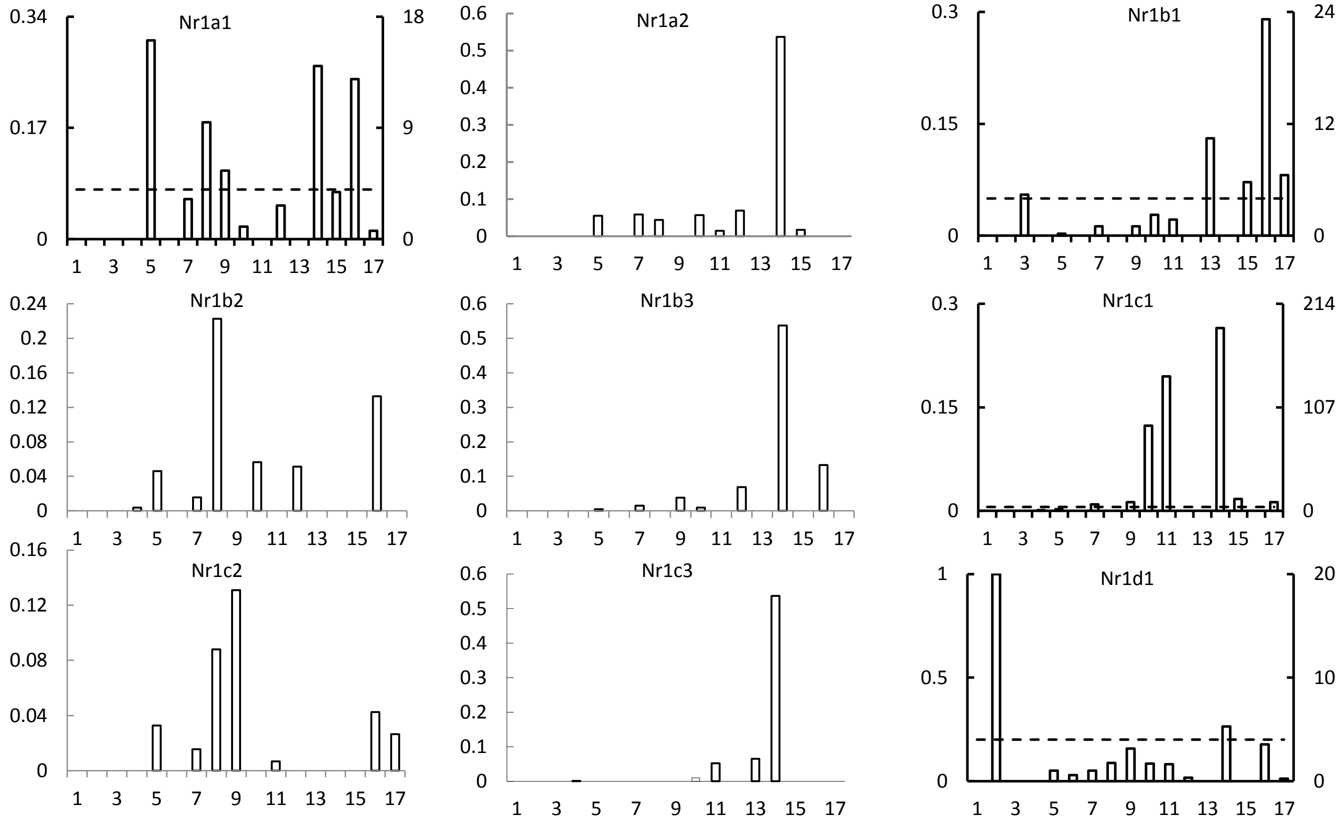

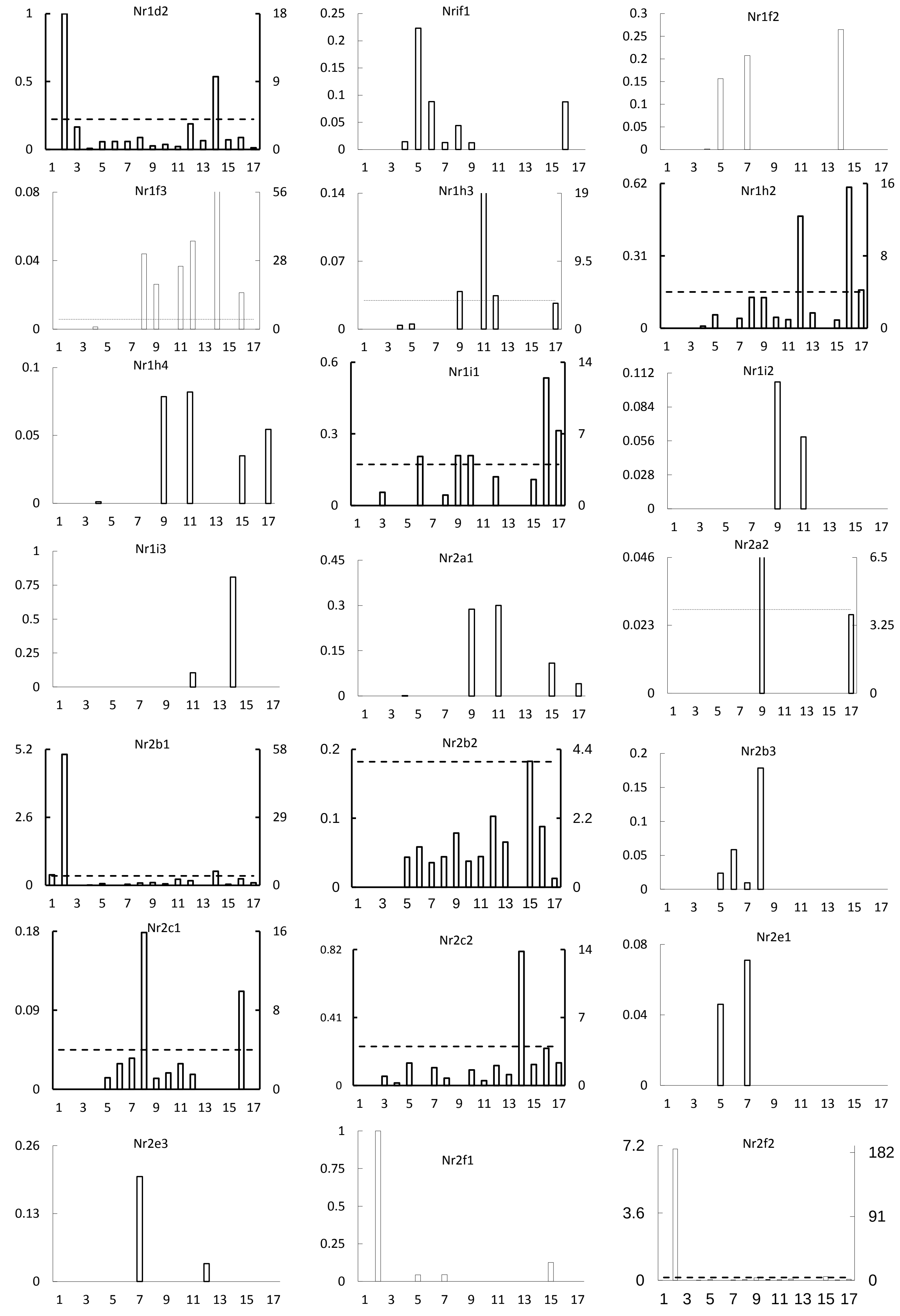
Nr1d2
Nrif1
Nr1f2
Nr1f3
Nr1h3
Nr1h2
Nr1h4
Nr1i1
Nr1i2
Nr1i3
Nr2a1
Nr2a2
Nr2b1
Nr2b2
Nr2b3
Nr2c1
Nr2c2
Nr2e1
Nr2e3
Nr2f1
Nr2f2

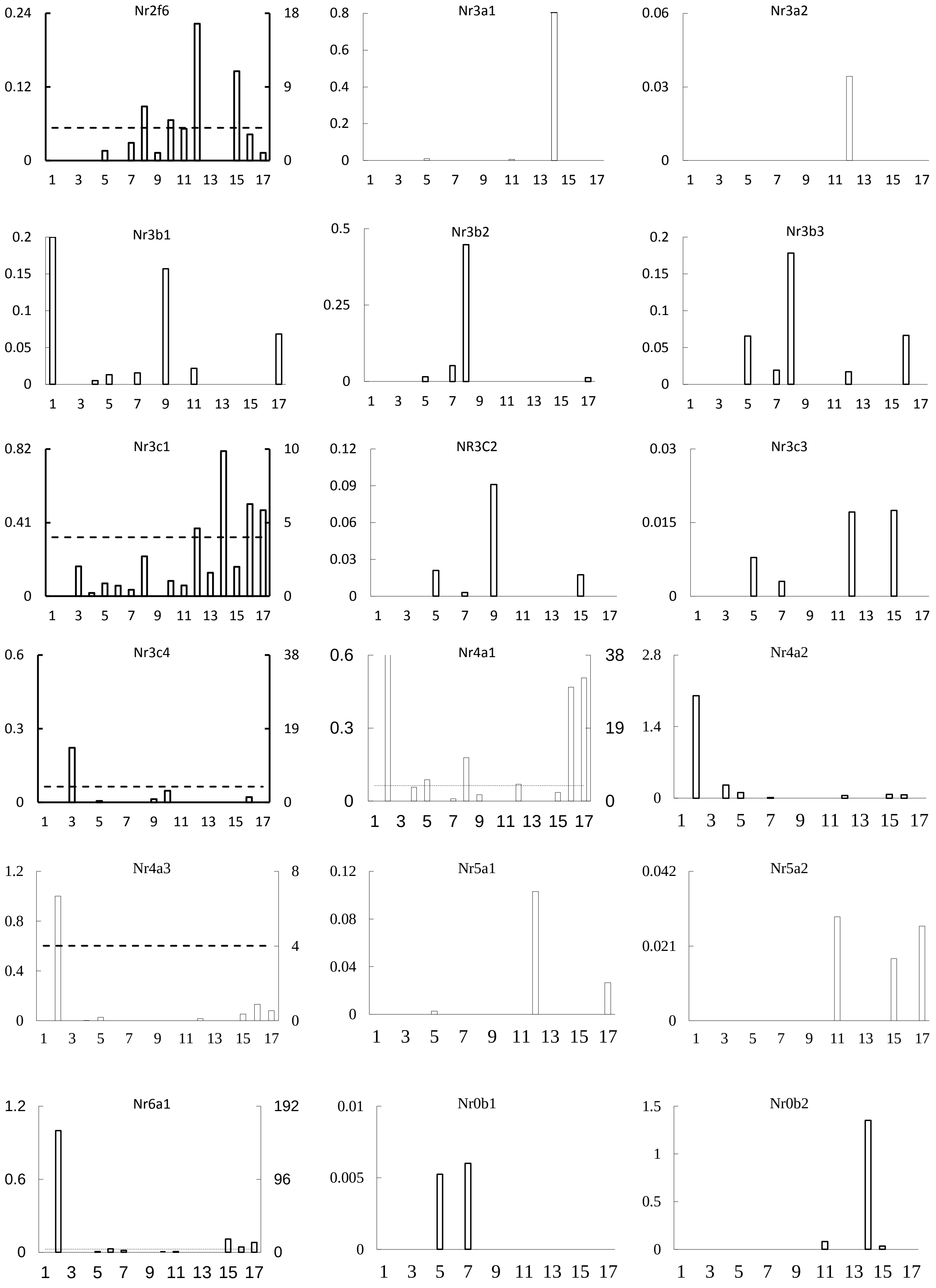

Nr2f6
0.24 0.12 0
18 9 0
1 3 5 7 9 11 13 15 17
Nr3a1
0.8 0.6 0.4 0.2 0
1 3 5 7 9 11 13 15 17
Nr3a2
0.06 0.03 0
1 3 5 7 9 11 13 15 17
Nr3b1
0.2 0.15 0.1 0.05 0
1 3 5 7 9 11 13 15 17
Nr3b2
0.5 0.25 0
1 3 5 7 9 11 13 15 17
Nr3b3
0.2 0.15 0.1 0.05 0
1 3 5 7 9 11 13 15 17
Nr3c1
0.82 0.41 0
10 5 0
1 3 5 7 9 11 13 15 17
NR3C2
0.12 0.09 0.06 0.03 0
1 3 5 7 9 11 13 15 17
Nr3c3
0.03 0.015 0
1 3 5 7 9 11 13 15 17
Nr3c4
0.6 0.3 0
38 19 0
1 3 5 7 9 11 13 15 17
Nr4a1
0.6 0.3 0
38 19 0
1 3 5 7 9 11 13 15 17
Nr4a2
2.8 1.4 0
1 3 5 7 9 11 13 15 17
Nr4a3
1.2 0.8 0.4 0
8 4 0
1 3 5 7 9 11 13 15 17
Nr5a1
0.12 0.08 0.04 0
1 3 5 7 9 11 13 15 17
Nr5a2
0.042 0.021 0
1 3 5 7 9 11 13 15 17
Nr6a1
1.2 0.6 0
192 96 0
1 3 5 7 9 11 13 15 17
Nr0b1
0.01 0.005 0
1 3 5 7 9 11 13 15 17
Nr0b2
1.5 1 0.5 0
1 3 5 7 9 11 13 15 17
