# Supplementary material for: A comprehensive data mining study shows that most nuclear receptors act as newly proposed homeostasis-associated molecular pattern receptors
Source: J Hematol Oncol. 2017 Oct 24;10:168. doi: 10.1186/s13045-017-0526-8 (PMC5655880; doi:10.1186/s13045-017-0526-8)
Supplement: Supplementary file 2 — Forty-eight nuclear receptors are associated with SAH and SAM/SAH ratio in mouse tissues. A: shows the correlation of 48 nuclear receptors associated with SAH levels (hypomethylation status) in mouse tissues. B. shows the correlation of 48 nuclear receptors associated with SAM/SAH ratio in mouse tissues. (PDF 648 kb) [file 13045_2017_526_MOESM2_ESM.pdf]

Supplementary figure 2. 48 nuclear receptors are associated with SAH and SAM/SAH ratio in mouse tissues.
A. 48 nuclear receptors are associated with SAH in mouse tissues.

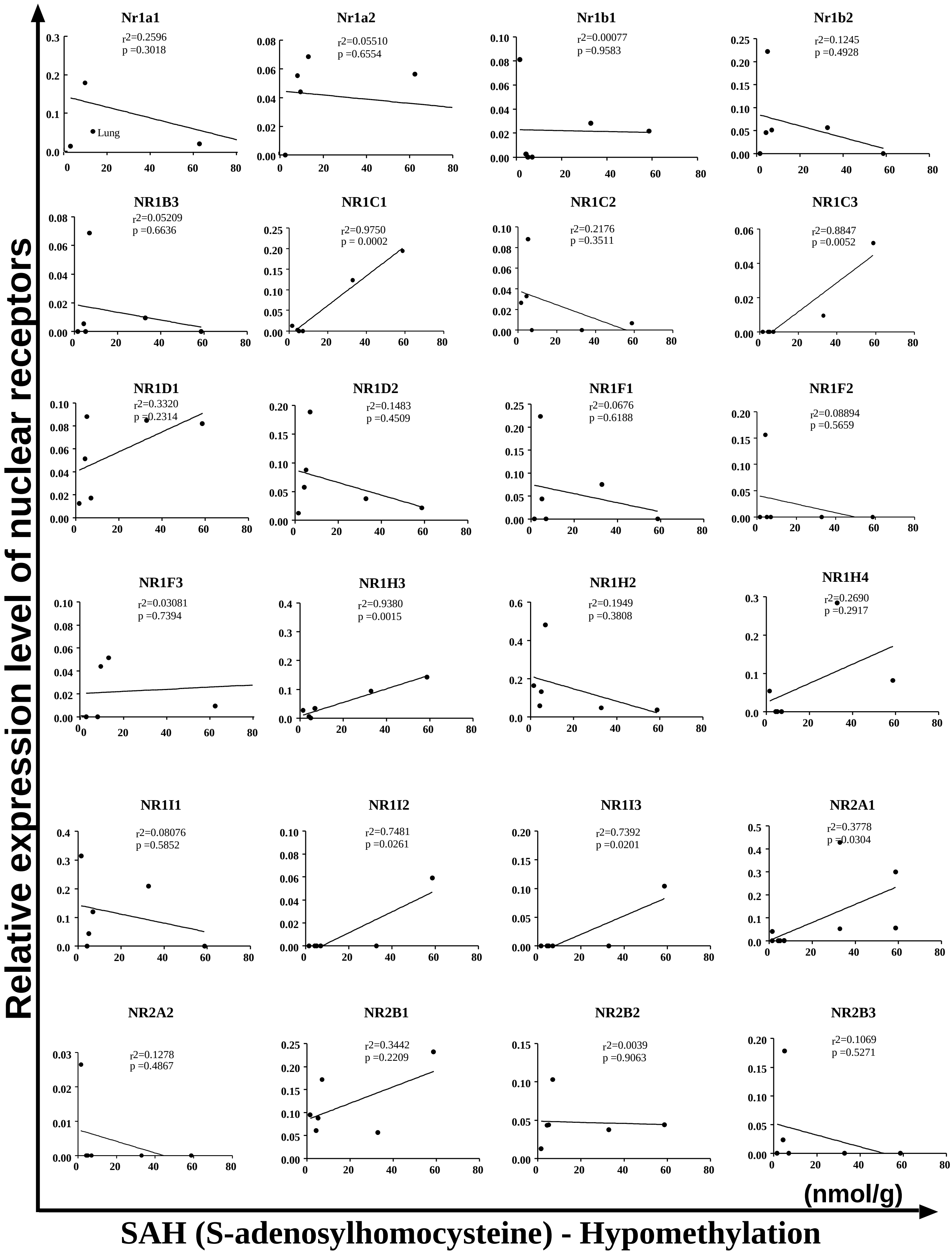

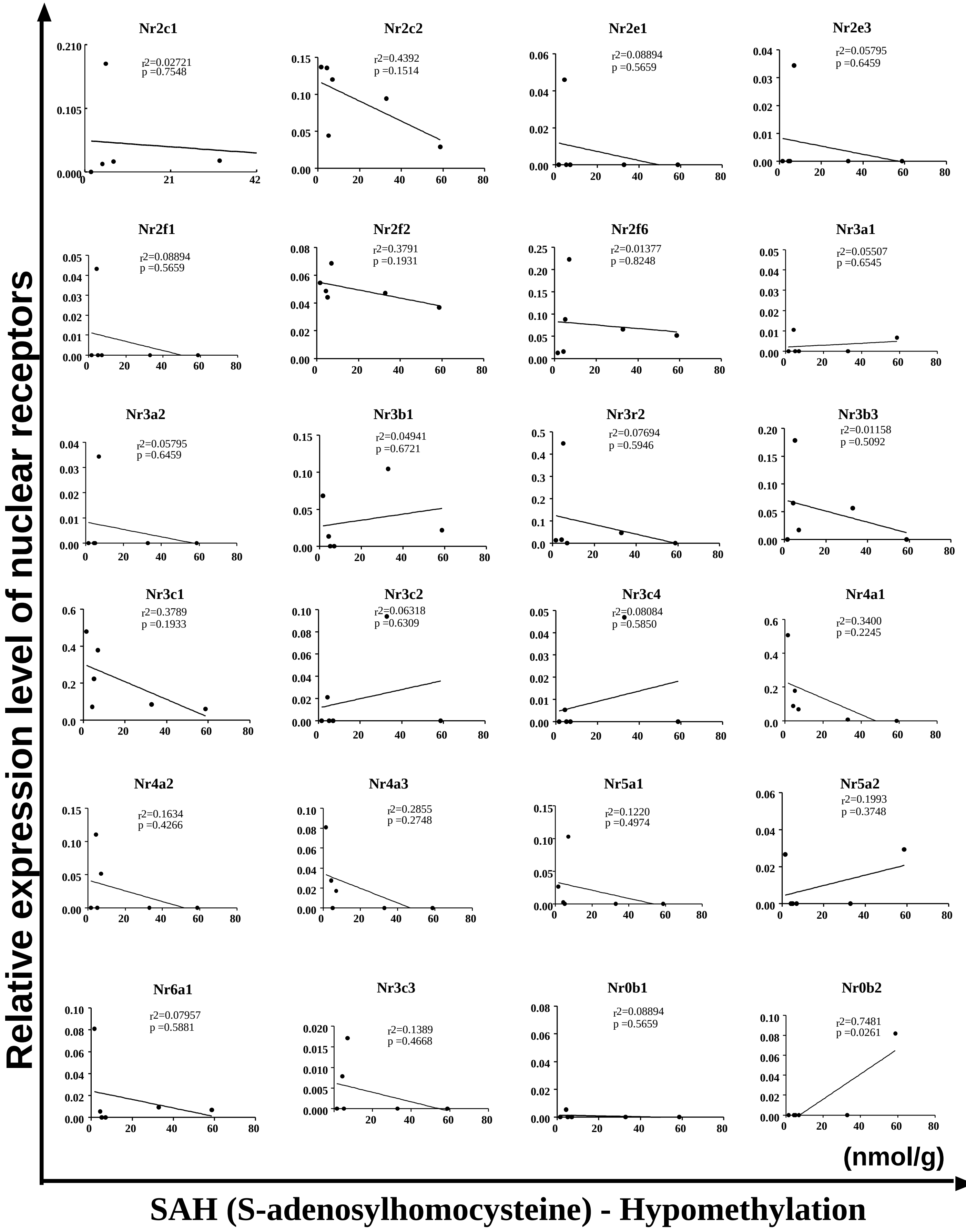

Relative expression level of nuclear receptors
Nr2c1
r2=0.02721
p =0.7548
Nr2c2
r2=0.4392
p =0.1514
Nr2e1
r2=0.08894
p =0.5659
Nr2e3
r2=0.05795
p =0.6459
Nr2f1
r2=0.08894
p =0.5659
Nr2f2
r2=0.3791
p =0.1931
Nr2f6
r2=0.01377
p =0.8248
Nr3a1
r2=0.05507
p =0.6545
Nr3a2
r2=0.05795
p =0.6459
Nr3b1
r2=0.04941
p =0.6721
Nr3r2
r2=0.07694
p =0.5946
Nr3b3
r2=0.01158
p =0.5092
Nr3c1
r2=0.3789
p =0.1933
Nr3c2
r2=0.06318
p =0.6309
Nr3c4
r2=0.08084
p =0.5850
Nr4a1
r2=0.3400
p =0.2245
Nr4a2
r2=0.1634
p =0.4266
Nr4a3
r2=0.2855
p =0.2748
Nr5a1
r2=0.1220
p =0.4974
Nr5a2
r2=0.1993
p =0.3748
Nr6a1
r2=0.07957
p =0.5881
Nr3c3
r2=0.1389
p =0.4668
Nr0b1
r2=0.08894
p =0.5659
Nr0b2
r2=0.7481
p =0.0261
(nmol/g)
SAH (S-adenosylhomocysteine) - Hypomethylation

Supplementary figure 2. 48 nuclear receptors are associated with SAH and SAM/SAH ratio in mouse tissues.
B. 48 nuclear receptors are associated with SAM/SAH ratio in mouse tissues.

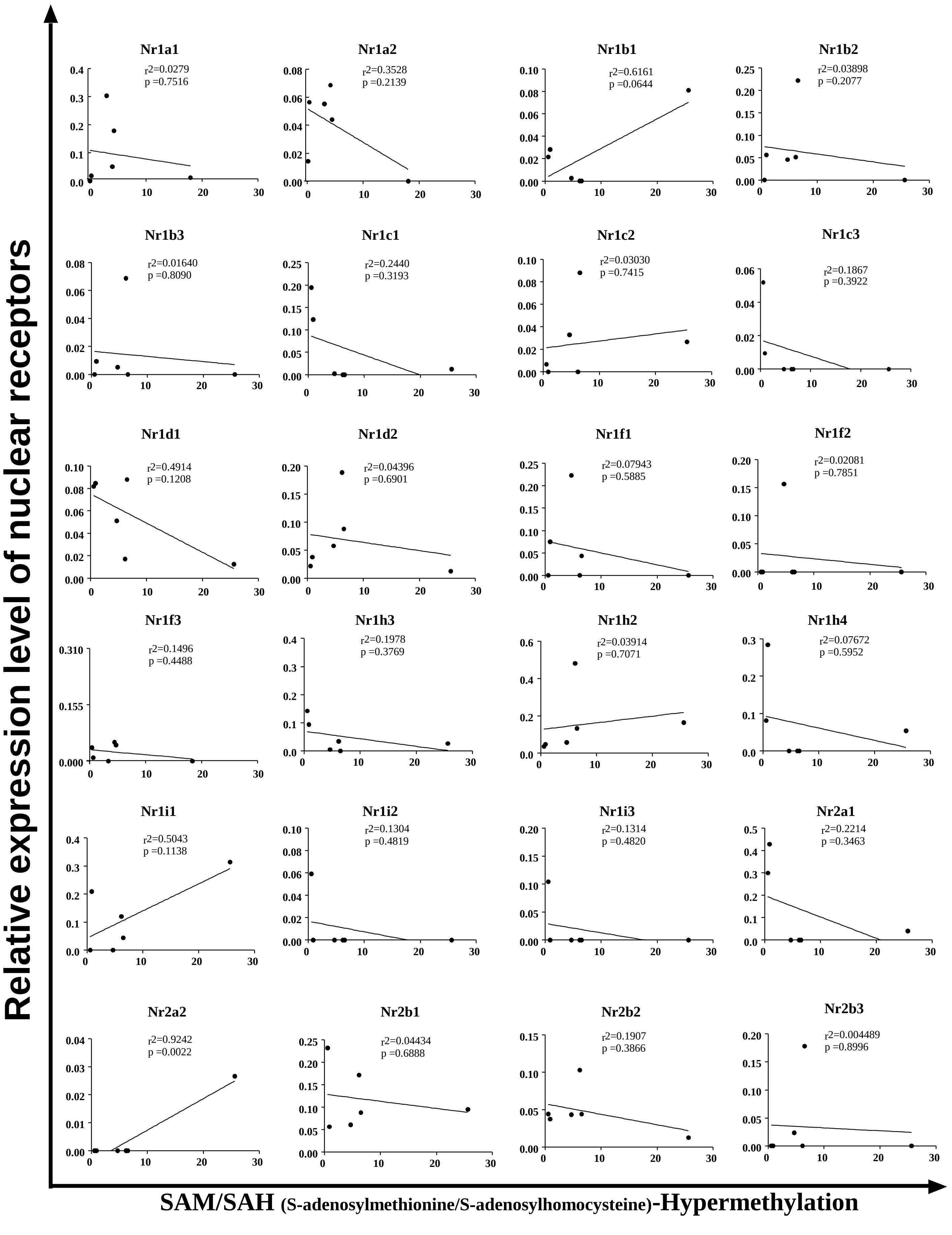

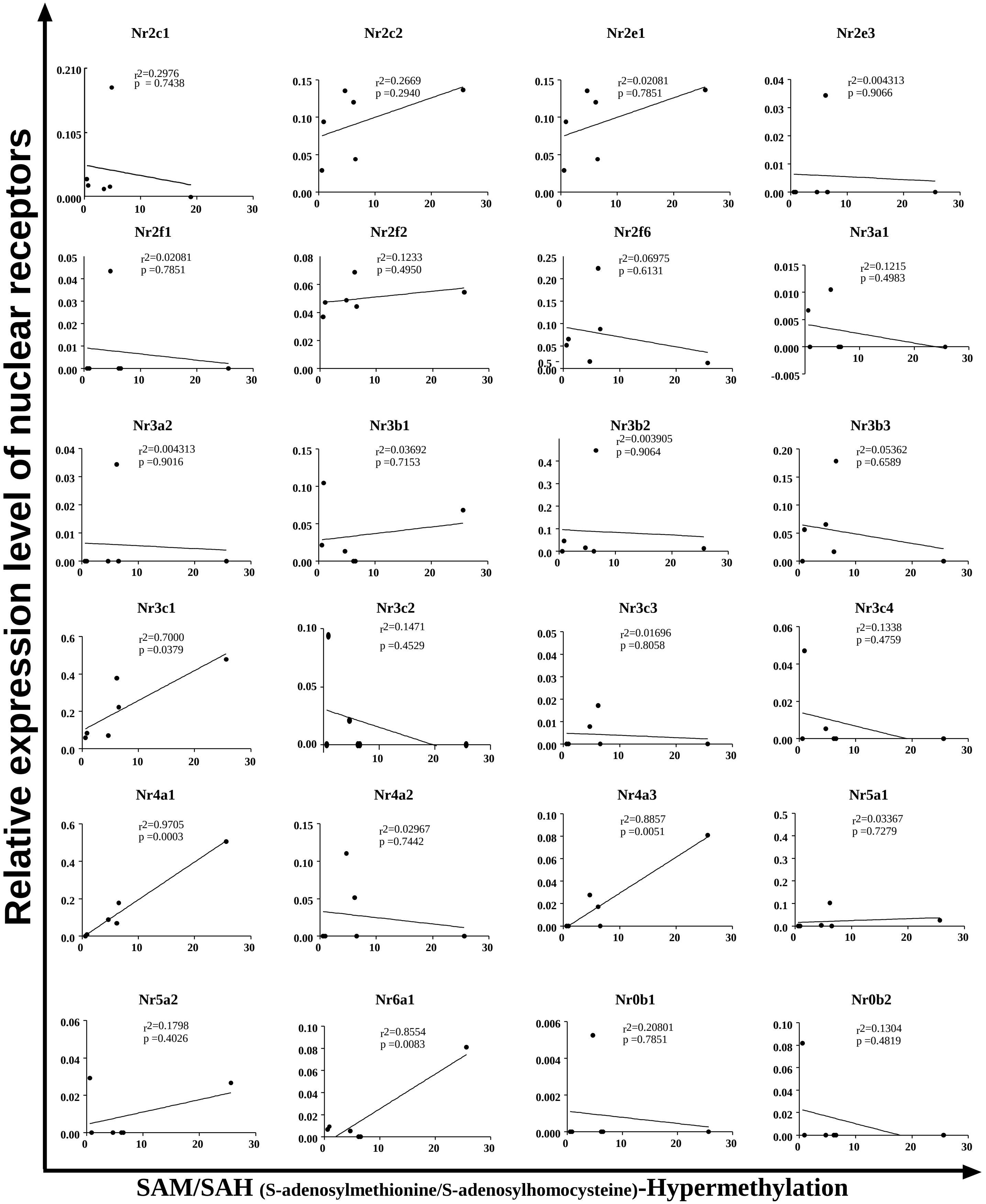

Relative expression level of nuclear receptors
Nr2c1
r2=0.2976
p = 0.7438
Nr2c2
r2=0.2669
p =0.2940
Nr2e1
r2=0.02081
p =0.7851
Nr2e3
r2=0.004313
p =0.9066
Nr2f1
r2=0.02081
p =0.7851
Nr2f2
r2=0.1233
p =0.4950
Nr2f6
r2=0.06975
p =0.6131
Nr3a1
r2=0.1215
p =0.4983
Nr3a2
r2=0.004313
p =0.9016
Nr3b1
r2=0.03692
p =0.7153
Nr3b2
r2=0.003905
p =0.9064
Nr3b3
r2=0.05362
p =0.6589
Nr3c1
r2=0.7000
p =0.0379
Nr3c2
r2=0.1471
p =0.4529
Nr3c3
r2=0.01696
p =0.8058
Nr3c4
r2=0.1338
p =0.4759
Nr4a1
r2=0.9705
p =0.0003
Nr4a2
r2=0.02967
p =0.7442
Nr4a3
r2=0.8857
p =0.0051
Nr5a1
r2=0.03367
p =0.7279
Nr5a2
r2=0.1798
p =0.4026
Nr6a1
r2=0.8554
p =0.0083
Nr0b1
r2=0.20801
p =0.7851
Nr0b2
r2=0.1304
p =0.4819
SAM/SAH (S-adenosylmethionine/S-adenosylhomocysteine)-Hypermethylation
